# Functional Autoantibodies Against G-Protein-Coupled Receptors: Insights into Molecular Mechanisms, Clinical Relevance and Therapeutic Perspectives

**DOI:** 10.3390/ijms27219475

**Published:** 2026-10-23

**Authors:** Ronja Zwerenz, Petra Lakatos, Merle Flecks, Gerd Wallukat, Bettina Hohberger

**Affiliations:** Department of Ophthalmology, Universitätsklinikum Erlangen, Friedrich-Alexander-Universität, Erlangen-Nürnberg, 91054 Erlangen, Germany; ronja.zwerenz@uk-erlangen.de (R.Z.); merle.flecks@uk-erlangen.de (M.F.);

**Keywords:** GPCRs, functional GPCR-targeting autoantibodies, agonistic and antagonistic autoantibodies, detection methods, therapeutic strategies

## Abstract

G-protein-coupled receptors (GPCRs) represent the largest superfamily of membrane-bound receptors in the human organism and are involved in the regulation of numerous physiological processes in almost all organ systems. Characterized by seven transmembrane domains and coupling to heterotrimeric G-proteins, they mediate signal transduction between extracellular stimuli and intracellular responses. Through these mechanisms they regulate critical processes such as cardiovascular function, neurotransmission, immune function, endocrine secretion, and metabolic homeostasis. Growing evidence suggests that functional autoantibodies directed against GPCRs (GPCR-fAAbs) may contribute to the pathophysiology of various diseases. By binding to extracellularly accessible regions of their target receptors, these antibodies can directly modulate receptor function and intracellular signaling. Depending on the receptor, epitope, and functional properties of the autoantibody, GPCR-fAAbs can act as agonists and activate receptor function or as antagonists and inhibit receptor function or alter the receptor signaling. Importantly, the presence of GPCR-directed autoantibodies does not necessarily indicate functional activity or pathogenicity, and their biological effects may depend on both autoantibody-specific and cellular factors. The potential role of GPCR-fAAbs has been described in a growing number of autoimmune and non-autoimmune diseases affecting various organ systems. This review article aims to provide a comprehensive overview of the current knowledge on GPCRs and GPCR-fAAbs focusing on their molecular mechanisms, functional characteristics, and potential pathophysiological roles in various diseases. Furthermore, current detection methods, clinical findings and established and emerging therapeutic strategies, including immunoadsorption (IA), aptamer-based neutralization, receptor blocking, and targeted B cell modulation, are critically discussed. Although all these approaches present certain challenges in their application, specific prospects for clinical implementation can be highlighted. Overall, GPCR-fAAbs represent an increasingly recognized pathophysiological factor with potential clinical implications, while their targeted therapeutic modulation remains an evolving area of translational research.

## 1. Introduction

With approximately 800 G-protein-coupled receptor (GPCR)-encoding genes, GPCRs are highly conserved and constitute the largest receptor family in the human genome [1]. They act as key mediators of signal transduction between extracellular stimuli and intracellular responses. GPCRs are classified into major families, including rhodopsin-like receptors (family A), the secretin receptor family (family B), and the metabotropic glutamate receptor family (family C) [1]. Beyond these classical subfamilies, the structural and functional diversity of the superfamily is further expanded by two distinct classes: the Frizzled (Class F) and the adhesion GPCR family (Class B2) [2,3]. The Frizzled receptor family comprises 10 members and the related smoothened receptor [2]. These receptors primarily bind Wnt glycoproteins through a highly conserved, extracellular N-terminal cysteine-rich domain [4]. The 33 human adhesion G-protein-coupled receptors (AGPCRs) link cell–matrix or cell–cell interactions with G-protein signaling [5]. AGPCRs are characterized by an exceptionally long extracellular N-terminus containing diverse adhesion modules and a GPCR autoproteolysis-inducing (GAIN) domain [6].

A common structural feature of all GPCRs is the presence of seven transmembrane domains connected by intracellular and extracellular loops (ECLs) [7,8]. While the physiological role of GPCRs is well established, it has become increasingly clear that dysregulated interactions with the immune system, particularly via autoantibodies directed against these receptors, may have pathophysiological or clinical relevance [9,10,11,12]. Functional autoantibodies directed against GPCRs (GPCR-fAAbs) can bind to extracellularly accessible regions of the receptor and directly modulate receptor function and signaling. ECLs have been identified as target structures for several GPCR-fAAbs, and the second extracellular loop (ECL II) contains important epitopes for some well-characterized autoantibodies [9,10]. Such antibody-mediated modulation of receptor function may therefore have a role in the pathophysiology of several diseases. Individual GPCRs show distinct tissue- and organ-specific expression patterns.

The regulation of the cardiovascular system involves adrenergic and muscarinic acetylcholine receptors, as well as GPCRs of the renin-angiotensin system. In the immune system, chemokine receptors play an important role in the migration and activation of leukocytes. Numerous GPCRs mediate the effects of neurotransmitters and neuromodulators in the central nervous system, while GPCRs in the lung, kidneys, and gastrointestinal tract regulate, among other functions, bronchial tone, fluid balance, and intestinal motility [13,14,15]. The diverse expression patterns and high functional diversity of GPCRs explain why alterations in GPCR signal transduction, for example through the binding of GPCR-fAAbs, can be associated with a variety of pathological conditions [16]. Figure 1 shows an exemplary illustration of a wide variety of receptors, assigned to human organs (see Figure 1).

## 2. GPCRs

### 2.1. Structural Characteristics of GPCRs

GPCRs are characterized by seven transmembrane domains, which form a characteristic helical structure within the lipid bilayer of the cell membrane [7]. The N-terminus is located extracellularly, while the C-terminus is located intracellularly. The length and structural characteristics of both vary depending on the receptor type [1]. Extracellularly accessible regions of the receptor provide potential binding sites for functional autoantibodies (fAAbs), while the intracellular domains are involved in signal transduction. The ECLs are particularly important in this context, especially ECL II, which represents an important autoantibody-binding region for several well-characterized GPCR-fAAbs [9,10]. However, other extracellular receptor regions, such as the N-terminal extracellular domain, may also serve as epitopes for GPCR-directed autoantibodies [17].

### 2.2. GPCR Signaling Pathways

Ligand binding induces a conformational change in the receptor, leading to activation of heterotrimeric guanosine triphosphate (GTP)-binding proteins (G-proteins). These proteins are composed of α-, β-, and γ-subunits. Upon activation, guanosine diphosphate (GDP) bound to the Gα subunit is exchanged for GTP, activating G-protein-mediated intracellular signaling pathways. A variety of downstream effectors are modulated by Gα and Gβγ, including adenylyl cyclase (AC), which regulates the production of cyclic adenosine monophosphate (cAMP). Further effectors include phospholipase C (PLC), which generates the second messengers inositol 1,4,5-trisphosphate (IP_3_) and diacylglycerol (DAG), as well as various ion channels [1,8]. Different intracellular signaling pathways are activated depending on the receptor subtype and associated G-protein family. Gi proteins inhibit AC, thereby reducing cAMP production, whereas Gs proteins stimulate AC, leading to increased intracellular cAMP levels and activation of protein kinase A (PKA). In contrast, Gq proteins activate phospholipase C beta (PLCβ), which produces DAG and IP_3_. DAG subsequently activates protein kinase C (PKC), which controls various cellular processes, such as gene transcription, cellular contraction, secretion, and proliferation, whereas IP_3_ induces the release of calcium from intracellular stores. GPCRs regulate a wide range of physiological activities in a highly cell-type-specific manner via the coordinated activation of multiple signaling cascades [1,8] (see Figure 2). Under physiological conditions, signal transduction is tightly regulated to prevent excessive activation and prolonged stimulation of the receptor. G-protein-coupled receptor kinase (GRK) phosphorylates the active receptor after prolonged agonist stimulation, thereby promoting the recruitment of β-arrestin. In addition to promoting receptor internalization and desensitization, β-arrestin can also activate G-protein-independent signaling pathways involved in receptor-specific physiological responses [7,18].

## 3. Functional Autoantibodies Against GPCRs

### 3.1. Structure, Subclass Classification, and Molecular Characterization

Many characterized autoantibodies directed against GPCRs belong to the immunoglobulin G (IgG) class and therefore share the characteristic IgG structure consisting of two identical heavy and two identical light chains. Each IgG molecule contains two fragment antigen-binding (Fab) regions and one fragment crystallizable (Fc) region. In general, the variable regions of the Fab domains mediate interactions with antigen recognition and binding, while the Fc region mediates interactions with Fc receptors, components of the complement system, and other elements of the immune system [19]. Human IgGs are classified into four subclasses: IgG1, IgG2, IgG3, and IgG4, which differ primarily in their constant regions, particularly in the structure of the hinge region and the second constant domain of the heavy chain (CH2) [20].

The IgG subclass characterization of GPCR-fAAbs has been reported for only a limited number of receptors [21]. For beta 1-adrenergic receptor (β1-AR) autoantibodies, Nagatomo et al. identified both IgG3 and non-IgG3 autoantibody populations, which were associated with different clinical outcomes in patients with cardiomyopathy [22]. In primary open-angle glaucoma, ocular hypertension and pseudoexfoliation-associated glaucoma, functionally active beta 2-adrenergic receptor (β2-AR) autoantibodies belonging to the IgG3 subclass have been identified [23,24]. In another well-characterized example, activating angiotensin II type 1 receptor (AT1R) autoantibodies identified in patients with renal-allograft rejection included the IgG1 and IgG3 subclasses [25]. These findings suggest that the subclass distribution of GPCR-directed autoantibodies may vary between receptor targets and disease conditions. Regarding their mechanisms of action, the functional effects of characterized GPCR-fAAbs have predominantly been attributed to binding of their antigen-binding regions to the target receptor. The subsequent modulation of receptor activity has been demonstrated experimentally for several GPCR-fAAbs, including autoantibodies against the thyroid-stimulating hormone receptor (TSHR) and β1- and β2-AR [17,26]. However, the different Fc-mediated effector properties of IgG subclasses should also be considered as additional mechanisms contributing to the biological effects of GPCR-fAAbs. Moreover, experimental studies in dilated cardiomyopathy (DCM) have demonstrated Fcγ receptor-dependent effects of autoantibodies, while clinical studies have further suggested that Fcγ receptor polymorphisms and IgG subclass may influence their biological or clinical effects [22,27]. In this context, IgG3 is characterized by strong Fcγ receptor interactions and complement-activating capacity, suggesting that these properties may also contribute to the biological effect of autoantibodies belonging to this subclass. The combined effects of receptor-specific modulation mediated by GPCR-fAAb binding and Fc-dependent immune effector mechanisms may contribute to the systemic biological consequences of GPCR-fAAbs beyond their direct modulation of the respective target receptor.

In-depth mechanistic studies and detailed structure-function analyses are required to determine the mechanisms by which antibodies activate GPCRs. The majority of work characterizing GPCR-fAAb function to date relies on polyclonal patient samples, which provide important evidence for receptor-specific activity but do not allow the contribution of individual autoantibody species to be distinguished [28]. Monoclonal antibodies generated by the immunization of different animal species have provided an important proof-of-principle that individual GPCR-directed antibodies can be linked to defined epitopes and receptor responses. For example, immunization with a stabilized β1-AR yielded monoclonal antibodies that bound diverse receptor epitopes and showed different antibody-receptor interactions reflecting those epitopes [29]. Furthermore, Wölfel et al. used monoclonal antibodies and epitope mapping to provide detailed information on critical amino acids within the ECL II region and its conformational requirements. In particular, the NDPK^211–214^ motif and the disulfide bond between Cys^209^ and Cys^215^ were identified as key structural features of the conformational autoepitope involved in autoantibody binding and β_1_-adrenoceptor activation [30].

The isolation of patient-derived monoclonal antibodies represents an important further step. In contrast to experimentally generated monoclonal antibodies, patient-derived monoclonal antibodies directly represent naturally occurring autoreactive B cell clones that have undergone antigen-driven selection and affinity maturation in affected individuals. They therefore provide a unique opportunity to link an individual antibody specificity to its epitope, receptor conformation, and functional activity. This principle has been successfully demonstrated for selected GPCR autoantibodies. For example, patient-derived human monoclonal antibodies targeting TSHR have enabled detailed functional and structural characterization of stimulatory and blocking antibody-receptor interactions [17,31,32].

### 3.2. Insights into Agonistic and Antagonistic Mechanisms

GPCR-fAAbs can modulate the activity of their target receptors in different ways. Several well-characterized GPCR-fAAbs are described in the literature as agonists, meaning that their binding to the receptor can lead to conformational changes and induce receptor activation and initiate intracellular signaling pathways. Experimental studies indicate that the effects of GPCR-fAAbs on their target receptors do not necessarily mirror those induced by the physiological receptor ligand. Depending on the target receptor and autoantibody, they may have partial agonistic activity, pathway-selective or biased signaling, and may also modulate receptor response to physiological or pharmacological agonists [33,34,35]. Furthermore, the functional outcome of autoantibody binding may depend on the epitope recognized by the GPCR-fAAb, the resulting changes in receptor conformation and signaling, as well as alteration in receptor desensitization and internalization [36,37].

Studies on some agonistic GPCR-fAAbs have shown that their binding to ECL II has been associated with their functional activity; however, this cannot be regarded as a universal rule. Indeed, autoantibodies directed against different ECLs of the same receptor may be present simultaneously without necessarily exerting equivalent modulatory effects on the receptor function [36,38] (see Figure 3). The extracellular epitope recognized by GPCR-fAAbs appears to depend on both the target receptor and the individual autoantibody. In class A GPCRs, the ECLs are located near the extracellular entrance of the ligand-binding pocket, with ECL II frequently contributing to ligand recognition and receptor activation. Autoantibody binding to these regions may therefore influence receptor conformation and signaling [39]. Structural studies using conformation-selective nanobodies and antibody fragments have demonstrated that antibody binding can stabilize distinct active or inactive GPCR conformations [40]. However, direct structural evidence for such conformational stabilization by naturally occurring GPCR-fAAbs remains limited. Moreover, functionally relevant epitopes are not restricted to the ECLs. For example, stimulating TSHR autoantibodies recognize the large N-terminal extracellular domain of the receptor [17].

Antagonistic GPCR-fAAbs have been less extensively studied than agonistic autoantibodies against GPCRs, although several examples have been reported. Such inhibitory effects have been described for autoantibodies against the muscarinic receptor in Sjögren’s syndrome. These autoantibodies reduced the functional response to the muscarinic acetylcholine M3 receptor (M3R) agonist carbachol in human salivary gland cells in vitro [41]. In another study, sera from five of nine patients with primary Sjögren’s syndrome and all of the six patients with secondary Sjögren’s syndrome inhibited carbachol-induced bladder contraction by approximately 50%. Sera from these patients also inhibited the effects of neuronally released acetylcholine at M3R [42].

As discussed above, the functional effects of GPCR-fAAbs cannot simply be divided into agonistic and antagonistic categories. For example, agonistic effects have been described for β2-AR-fAAbs and inhibitory effects for M3R-fAAbs, while β1-AR-fAAbs have been reported to exhibit partial agonistic as well as pathway-selective signaling effects [33,35]. In addition, enhanced effects of pharmacological agonists in the presence of β2-AR-fAAbs have been reported in a study [16]. Therefore, characterization of their functional properties may require multiple experimental models and complementary readouts to capture the complexity of GPCR-fAAb-mediated receptor modulation [36,37].

### 3.3. Aspects of Origin, Functional Activity and Pathogenic Relevance

The presence of autoantibodies does not necessarily indicate a pathological condition. Autoreactive antibodies are part of the normal immune repertoire and can contribute to different immunoregulatory processes [43]. Autoantibodies against GPCRs have also been detected in healthy individuals in several studies, although their functional activity was not consistently confirmed by in vitro experiments. Cabral-Marques et al. described interconnected networks of GPCR-directed autoantibodies and demonstrated functional activity for selected autoantibodies, including effects on cell migration [37,44].

The mechanisms underlying the development of GPCR-directed autoantibodies are not fully understood and several possible mechanisms have been proposed [12,45]. Following infection, several mechanisms may contribute to the development of autoreactive immune responses. These include molecular mimicry, bystander activation, epitope spreading, and the enhanced exposure of self-antigens during tissue damage. Although these processes are well recognized in post-infectious immune responses, there is currently no direct evidence as to which of them are responsible for the development of GPCR autoantibodies [46,47].

Also, tissue damage resulting from inflammation can expose normally inaccessible epitopes to the immune system. This damage can occur through several mechanisms and includes epitopes present on GPCR-containing membrane fragments. Additionally, the increased levels of inflammatory mediators that accompany tissue damage can lead to bystander activation. Through this mechanism, a small pre-existing population of autoreactive B cells that recognize an epitope on a GPCR can become activated and multiply, resulting in increased production of the corresponding autoantibodies. Epitope spreading has likewise been proposed as a mechanism that may contribute to the development of GPCR-directed autoantibodies. These processes may act individually or in combination, which could be particularly relevant in the context of persistent infection and chronic inflammation [48,49].

Chagas disease is a parasitic infection caused by *Trypanosoma cruzi* that can lead to cardiomyopathy. In a well-documented example of molecular mimicry, Ferrari et al. showed that an epitope of the parasite’s P0 ribosomal protein shares a short homologous sequence with ECL II of the β1-AR. These cross-reactive antibodies were found to increase the beating rate of primary cardiomyocytes in a bioassay, an effect that could be inhibited by a specific β1-AR blocker. This demonstrates how molecular mimicry can contribute to the development of functional GPCR autoimmunity [50].

Nevertheless, functional activity alone does not necessarily imply pathogenicity. Whether a GPCR-fAAb contributes to disease development may depend not only on its concentration in vivo, but also on the local tissue microenvironment, including inflammatory, metabolic, and hypoxic conditions [51,52,53,54]. However, it could be shown that functional activity of GPCR-fAAb is significantly enhanced in inflammatory or ischemic regions (as shown for AT1R-fAAb) [55].

Jahns and colleagues immunized rats with the ECL II of the β1-AR, which induced the generation of agonistic functional autoantibodies against beta 1 (β1-fAAbs). After approximately nine months, the animals developed progressive left ventricular dilatation and deterioration of cardiac function. Particularly important evidence for pathogenicity came from the repeated transfer of serum from autoantibody-positive animals to syngeneic healthy rats, which induced a similar cardiomyopathic phenotype. These findings provided experimental evidence that receptor-stimulating β1-fAAbs can themselves contribute to the progressive deterioration of cardiac function [38].

In humans, evidence for a pathogenic role of GPCR-fAAbs has been particularly well documented for agonistic autoantibodies against AT1R in preeclampsia. In 1999, Wallukat et al. first demonstrated that IgG from patients with preeclampsia could activate the AT1R in a cardiomyocyte bioassay and identified the ECL II of the receptor as a relevant epitope [56]. Subsequently, Thway et al. provided further evidence for the functional activity of these autoantibodies by demonstrating increased intracellular Ca^2+^ mobilization through AT1R activation [57]. Importantly, Zhu et al. later demonstrated that administration of IgG from women with preeclampsia or affinity-purified AT1R autoantibodies to pregnant mice induced key features of preeclampsia, including hypertension and proteinuria. This provided direct in vivo evidence for their pathogenic potential [58]. Further clinical evidence also showed a high prevalence of AT1R agonistic autoantibodies in preeclampsia and an association between autoantibody levels and disease severity, including blood pressure and proteinuria [59].

## 4. Detection and Functional Characterization

The detection of autoantibodies against GPCRs involves two different aspects. On the one hand, their binding to their target receptor can be assessed; on the other hand, characterizing their functional effects can be a major challenge. For this reason, various detection methods are combined to assess autoantibody binding, functional activity, and other important characteristics. However, methodological standardization remains a challenge in the field. Differences in assay design, experimental conditions, and criteria used to define autoantibody positivity or functional activity can limit comparability between studies. Furthermore, sample characteristics may vary between laboratories because sample preparation is not standardized, and autoantibody levels can be influenced by several factors, including age, sex, and comorbidities. As previously mentioned in the context of investigating molecular mechanisms, the use of widely available monoclonal antibodies could also facilitate the standardization of detection methods. These antibodies could serve as standardized reference materials for comparing different approaches across laboratories and identifying suitable methods for subsequent standardization. However, it should be acknowledged that monoclonal antibodies may not fully reflect the characteristics of antibodies present in patient samples.

In addition, functional GPCR-fAAb assays have been established and applied by a relatively limited number of laboratories, emphasizing the need for independent replication, interlaboratory validation, and standardized reference materials before these assays can be broadly implemented in clinical diagnostics. The main methodological approaches currently used for the detection and functional characterization of GPCR-directed autoantibodies, together with their respective advantages and limitations, are discussed in the following sections.

### 4.1. Cardiomyocyte Bioassay

Since GPCR-fAAbs are characterized by their ability to modulate receptor function, functional bioassays are required to assess their receptor-mediated effects [11].

Among these specific bioassays, one of the longest-established and most widely published methods is the cardiomyocyte bioassay developed by Wallukat and Wollenberger [60]. In this assay, primary cardiomyocytes forming spontaneously and synchronously beating clusters are used. They are isolated from the hearts of neonatal rats and serve as biological sensors for GPCR-fAAb-mediated functional effects. These primary cells express several functional receptors, including adrenergic, muscarinic, and endothelin receptors. They respond sensitively to receptor-specific stimuli, including agonists, antagonists, and autoantibodies targeting these receptors [54,61].

In this procedure, IgG fractions are purified from patient sera using different methods and are added to the cultured cardiomyocytes. The whole purification process aims, among other purposes, to remove low-molecular-weight, potentially biologically active serum components, thereby reducing possible non-IgG-mediated effects. In the protocol used by Wallukat et al., for example, the IgG fraction was isolated by ammonium sulfate precipitation, followed by dialysis to remove low-molecular-weight components [54].

Following the addition of the IgG fraction to the cell culture, changes in the spontaneous beating rate can be monitored over time and quantified manually or digitally relative to the baseline frequency. Depending on the physiological function of the target receptor, GPCR-fAAbs can induce either positive or negative chronotropic responses. For example, agonistic autoantibodies against β1-AR or β2-AR increase the beating rate of spontaneously contracting rat cardiomyocytes, resulting in a positive chronotropic effect. In contrast, agonistic autoantibodies against the muscarinic acetylcholine M2 receptor (M2R) decrease the beating rate and therefore exert a negative chronotropic effect [38,60]. Since neonatal cardiomyocytes express several different GPCRs, the receptor specificity of the observed functional response must be determined experimentally. By using receptor-specific antagonists, the effect mediated through the respective receptor can be blocked. Therefore, a reduction or complete neutralization of the GPCR-fAAb-induced change in beating rate allows the functional activity to be assigned to the respective GPCR [25,62]. This approach has been demonstrated using specific antagonists for several receptors, including β2-AR, alpha1-adrenergic receptor (α1-AR), AT1R, endothelin A receptor (ETAR), Mas receptor (MasR), and M2R [54].

The limitations of the cardiomyocyte bioassay include the considerable technical effort required, the need for repeated preparation of primary neonatal rat cardiomyocytes, the associated biological variability, and low throughput. Despite these limitations, alternative methods have not yet clearly replaced the classical cardiomyocyte bioassay for the investigation of GPCR-fAAbs [12].

### 4.2. Binding-Based Assays

Enzyme-linked immunosorbent assay (ELISA) is a widely used method for the detection of autoantibodies against GPCRs. For detection, synthetic linear or cyclic peptides representing extracellular regions of different GPCRs, as well as recombinant receptors or receptor-containing membrane preparations in some assays, are used as antigens [10,63].

Using an appropriate standard or calibration curve, ELISA allows quantitative or semi-quantitative determination of antibody reactivity. The method is relatively simple and rapid to perform and can be performed directly using serum without prior purification of the IgG fraction. Consequently, ELISA-based approaches have been widely used for the detection of GPCR-directed autoantibodies in recent studies, particularly in the context of Long-COVID [16,64,65]. However, an important limitation of ELISA is that it detects antibody binding to the respective antigen but does not provide direct information about the functional activity of the antibodies. Thus, ELISA positivity indicates the presence of antibodies binding to the applied receptor antigen. However, it does not necessarily demonstrate that these autoantibodies can activate or inhibit the native receptor [66].

A further limitation of this method is that synthetic peptides do not necessarily reproduce the native three-dimensional conformation of the extracellular receptor regions. Consequently, autoantibodies recognizing conformation-dependent epitopes may not be detected by peptide-based assays. Furthermore, binding to a linear peptide does not necessarily indicate recognition of the native receptor or the ability to modulate its function [28,30]. For β1-AR autoantibodies, for example, it has been shown that only a subset of autoantibodies reacting with receptor-derived peptides recognized the native human receptor expressed in the cell membrane. This subset also showed receptor-activating functional activity, as assessed by cAMP production [66].

### 4.3. Alternative Cell-Based Assays

As receptor binding alone does not establish biological activity, characterization of functional GPCR-fAAbs requires assays that measure changes in receptor signaling or receptor-dependent cellular responses like alterations in intracellular second messengers [28,67,68]. Different functional readouts can be used depending on the receptor and its signaling pathway. These include cAMP, intracellular Ca^2+^, β-arrestin recruitment, and receptor internalization, among other downstream processes. Such approaches can distinguish the stimulatory, inhibitory, and pathway-selective effects of GPCR-fAAbs. Single reports using alternative cell-based assays are available in the literature, yet no specific method has been established as the gold-standard to date.

One molecular approach is based on a cAMP-based assay. For instance, the activity of α1-, β1- and β2-ARs in patient-derived IgGs has been tested in cells expressing the receptors using immunoassays or fluorescence resonance energy transfer (FRET)-based approaches. They serve to quantify changes in intracellular cAMP or the conformational change of β-lactamase induced by cAMP. Furthermore, reporter-based systems, including cAMP-responsive luciferase assays and genetically encoded cAMP biosensors, enable the detection of receptor-mediated responses more rapidly and homogeneously. These methods have also been used to study GPCR autoantibodies, including TSHR autoantibodies [69].

Ca^2+^-related assays provide an additional approach. For example, AT1R autoantibodies were functionally characterized in AT1R-expressing Huh7 cells by quantifying changes in intracellular Ca^2+^ using an aequorin-based sensor. The response was inhibited by the AT1R antagonist losartan, supporting receptor-specific activity [70,71].

Following the discovery of G-protein-independent β-arrestin signaling, functional assays have been developed to detect β-arrestin recruitment to GPCRs or β-arrestin-induced GPCR internalization [72]. In addition to the classic chemiluminescence-based assay systems [35,73], multiple functional assays using resonance energy transfer (RET) have been developed in recent years to directly monitor conformational changes in GPCRs, G-proteins and β-arrestin. These sensors have been used to investigate GPCR signaling profiles and the GPCR activation process at multiple scales [72,74]. For instance, sera from Postural Orthostatic Tachycardia Syndrome (POTS) patients were analyzed using a FRET-based reporter system involving a β2-arrestin transcription factor that drives the transcription of a β-lactamase gene [75].

Combining different functional readouts can provide additional mechanistic information. In β1-AR autoimmunity, for example, Mohan et al. showed that IgG3-positive β1-AR autoantibodies reduced agonist-induced AC activity and cAMP generation, but increased β-arrestin recruitment and ERK activation through phosphorylation. This suggests pathway-selective or biased signaling [35]. Similarly, Bornholz and colleagues combined measurements of β1-AR conformation, cAMP production, and receptor internalization. They found that autoantibody-induced receptor conformational changes did not always correlate with cAMP stimulation, while some antibodies also attenuated receptor internalization. These findings demonstrate that antibody-induced receptor conformational changes do not necessarily result in uniform receptor activation, suggesting that the functional effects of GPCR-fAAbs cannot be fully captured by a single downstream readout [36].

Overall, cell-based functional assays demonstrate receptor-mediated activity beyond antibody binding. However, their results may depend on the cellular system, receptor expression level, assay conditions, and the selected downstream readout, which can limit comparability across studies. Furthermore, investigating the long-term effects of GPCR-fAAb, such as receptor desensitization, internalization and adaptive changes in downstream signaling, require additional approaches.

## 5. Functional GPCR Autoantibodies in Human Diseases

GPCR expression patterns and the occurrence of associated autoantibodies have been investigated in a broad range of clinical conditions. Although fAAbs currently do not appear to be disease-specific, certain autoantibodies are more frequently associated with particular clinical manifestations [76]. Furthermore, sex- and age-related differences in GPCR autoantibody profiles have been reported in healthy individuals, while sex-specific associations have also been observed in a large clinic-based cohort [44,77]. However, it remains unclear whether diseases with pronounced sex- or age-related distributions are accompanied by corresponding differences in GPCR-fAAb positivity or functional activity. Most available studies were not specifically designed to systematically address this question.

GPCR-fAAbs are associated with a variety of autoimmune and non-autoimmune diseases affecting different organ systems. Associations between the presence of specific GPCR-fAAbs and particular clinical manifestations have been reported across various diseases, as summarized in Table 1.

## 6. Therapeutic Approaches

In certain diseases, GPCR-fAAbs may contribute to pathophysiological processes through their effects on receptor function and therefore represent potential targets for therapeutic intervention [45,51,104]. Current therapeutic concepts include the physical removal of circulating autoantibodies, their molecular neutralization, and pharmacological inhibition of GPCR-fAAb-mediated receptor signaling. In addition, promising strategies aim to reduce autoantibody production by targeting the underlying immune mechanisms. These approaches differ considerably in terms of their mechanisms, clinical evidence, and limitations. Several of these applications are currently in the experimental or early-stage clinical development phase (see Figure 4).

### 6.1. Immunoadsorption

Immunoadsorption (IA) is a specific apheresis procedure in which circulating immunoglobulins, including potentially pathogenic autoantibodies, are removed from the patient’s plasma. The procedure is based on passing the separated plasma through an adsorption column, where immunoglobulins are bound and removed before it is returned to the patient’s body (see Figure 4).

In DCM, autoantibodies against β1-AR have been considered to be of pathophysiological relevance. Accordingly, several studies have investigated the use of IA and the potential therapeutic benefit of removing circulating autoantibodies [21,105].

Felix et al. showed that IgGs isolated from the IA eluates of patients with DCM exhibited cardiodepressive activity. Patient-derived IgGs reduced Ca^2+^ transients and cardiomyocyte contractility, whereas IgG from healthy controls produced no comparable functional effects. Although the study did not identify the specific autoantibodies responsible for these effects, the findings suggested that circulating autoantibodies may contribute to myocardial dysfunction in DCM [106].

In a prospective case–control study, Müller et al. investigated the long-term effects of IA treatment in 34 β1-AR-fAAb-positive idiopathic DCM patients. All of them received standard medical therapy, but 17 patients in the cohort subsequently underwent IA as well. In these patients IA resulted in the removal of β1-AR-fAAb, which was undetectable during the one-year follow-up period. Furthermore, in the IA-treated group an increase in left ventricular ejection fraction was observed, accompanied by a reduction in left ventricular end-diastolic diameter. In contrast, in the control group there was no significant improvement in left ventricular function [107].

However, the beneficial effects of IA in DCM may not be exclusively attributable to the removal of β1-AR-fAAbs. Mobini et al. investigated the effects of IA in 22 patients with DCM together with the detection of β1-fAAbs using several methods, including ELISA and a functional cardiomyocyte bioassay. Overall, 72% of the patients were positive for β1-fAAbs. IA treatment was associated with acute and prolonged hemodynamic improvement as well as increased left ventricular ejection fraction in both β1-fAAb-positive and -negative patients. Since the beneficial effects of IA were not restricted to β1-fAAb-positive patients, the authors concluded that the effects of IA in DCM could not be explained solely by the removal of these autoantibodies. They further concluded that other factors removed during IA may also contribute to the observed clinical effects [108].

Dandel et al. subsequently investigated the long-term clinical effects of IA therapy in β1-fAAb-positive patients with end-stage DCM. Removal of β1-fAAbs by either specific or non-specific IA was associated with improved cardiac function and long-term clinical stabilization. Treated patients also showed a more favorable heart transplantation or ventricular assist device-free survival. Furthermore, the reappearance of β1-fAAbs was associated with recurrent deterioration of cardiac function in 76% of cases [109]. These findings support an association between β1-fAAb removal and the long-term clinical benefits observed after IA, while the findings of Mobini et al. suggest that additional mechanisms may also contribute to the therapeutic effects of IA. More recently, the randomized, double-blind, sham-controlled IASO-DCM trial including 171 patients found no significant benefit of IA followed by immunoglobulin G substitution (IA/IgG) over sham treatment regarding left ventricular ejection fraction improvement or heart failure symptoms at six months. The key composite secondary endpoint was also similar between the groups. However, subgroup analyses, including those involving patients with cardiac autoantibodies, were limited by small sample sizes and require further investigation [110,111].

The potential benefits of IA therapy have also been investigated in glaucoma. Jünemann et al. detected functionally active GPCR autoantibodies in approximately 75% of patients with open-angle glaucoma or ocular hypertension, whereas such activity was not detected in healthy controls. Among the GPCR-fAAbs investigated, agonistic autoantibodies targeting the β2-AR were of particular relevance. In a subsequent small proof-of-principle study, four patients with primary open-angle glaucoma received IA treatment. Following IA, functional autoantibodies against beta 2 (β2-fAAbs) were no longer detectable using the cardiomyocyte bioassay, together with a decrease in intraocular pressure. Given the small number of patients included in this proof-of-principle study, further studies are needed to evaluate the therapeutic potential of IA in glaucoma [23].

However, IA has several limitations that complicate its widespread clinical application. The current adsorption systems are generally not specific to individual potential pathogenic autoantibodies. This means that, along with the targeted GPCR-fAAbs, other circulating immunoglobulins are also removed [19,20]. In some IA protocols, intravenous immunoglobulin substitution has been administered after treatment, which may further increase the therapeutic burden and costs. Furthermore, IA is an invasive and resource-intensive procedure that may require repeated treatment sessions and close clinical monitoring [112].

### 6.2. Aptamers

One potential approach for neutralization of functionally active autoantibodies against GPCRs is the use of aptamers (see Figure 4). Aptamers are single-stranded oligonucleotides that can bind to specific target molecules with high affinity [104]. The synthetic DNA aptamer BC007 (rovunaptabin) has been shown to neutralize GPCR-fAAbs in several in vitro and in vivo studies. Its mechanism of action is based on binding to these GPCR-fAAbs and preventing their interaction with the respective receptors, thereby inhibiting their functional and potentially pathogenic effects [113].

BC007 was originally selected as a thrombin inhibitor from a group of similar aptamers, but its development as an anticoagulant was not continued due to its very short duration of action [114]. It later gained renewed attention when Wallukat et al. demonstrated that the aptamer was able to neutralize β1-fAAbs isolated from patients with dilated cardiomyopathy in vitro, as determined using the cardiomyocyte bioassay [115]. Further studies indicated that the effect of BC007 was not limited to a single type of autoantibody. Rather, the aptamer was able to bind to and neutralize the receptor-activating effects of GPCR-fAAbs, including β1-AR, β2-AR, α1-AR, ETAR, and AT1R [116].

Based on these preclinical findings, Becker et al. published the results of a three-part Phase I study investigating the safety, tolerability, pharmacokinetics, and mode of action of BC007 in healthy subjects. While the initial part primarily assessed safety and tolerability in GPCR-fAAb-negative participants, subsequent studies included healthy but GPCR-fAAb-positive subjects to investigate the autoantibody-neutralizing effect of BC007. Neutralization showed a dose-dependent trend, and at the highest dose investigated, the detected GPCR-fAAbs were neutralized in all treated participants. No serious treatment-related adverse events were observed; however, a transient prolongation of activated partial thromboplastin time was reported [117].

More recently, BC007 has been investigated as a potential therapeutic approach in patients with Post-COVID syndrome. Hohberger et al. conducted a prospective, exploratory, placebo-controlled, double-blind, randomized Phase IIa trial in patients with Post-COVID syndrome and GPCR-fAAbs. In addition to neutralization of GPCR-fAAbs, the study reported statistically significant group-level improvements in several secondary patient-reported outcomes related to fatigue in the treatment group compared with placebo. Significant group-level improvements were also reported in selected SF-36 measures, including vitality, general health, social functioning, and the mental component summary. However, improvements were not observed consistently across all outcome measures or in all individual patients. These proof-of-concept findings should be interpreted in the context of the exploratory nature of the study, including small sample size and the limited follow-up period [118].

Subsequently, according to sponsor-reported topline results from the larger randomized, double-blind, placebo-controlled Phase II BLOC trial (NCT05911009; *n* = 119), BC007 did not demonstrate superiority over placebo for the clinical primary endpoint. The trial separately assessed clinical efficacy and GPCR-fAAb neutralization; however, detailed results, including the complete target-engagement data, have not yet been published. The sponsor reported that BC007 was safe and well tolerated [119].

### 6.3. Pharmacological Receptor Blockade

Conventional therapeutic approaches aim to interrupt or inhibit GPCR-fAAb-induced receptor signaling pathways [62] (see Figure 4). The clinical efficacy of β-blockers in the treatment of heart failure has been increasingly well documented. In dilated cardiomyopathy, β-AR blockers are used to reduce the persistent receptor stimulation caused by agonistic autoantibodies through competitive inhibition of β1-AR [38,120,121]. Furthermore, AT1R blockers are used in diseases such as systemic sclerosis or renal allograft rejection [25,101].

Pharmacological inhibition of the target receptor can reduce downstream signaling, but does not eliminate or neutralize the autoantibodies themselves or prevent their continued presence in circulation [62]. For this reason, these types of therapies are limited in their efficacy and can only contribute to the symptomatic management of the diseases, rather than providing a curative approach [11,12].

### 6.4. Perspectives for Future Therapeutic Development

Future therapeutic approaches could potentially combine tissue-targeting peptides with aptamer-based strategies to improve the local delivery and specificity of autoantibody-directed therapies [122,123]. In Long-COVID and myalgic encephalomyelitis/chronic fatigue syndrome (ME/CFS), combined therapeutic approaches involving IA followed by intravenous immunoglobulin administration have also been discussed as a potential strategy for modulating autoantibody-mediated processes [124].

One potential therapeutic approach is to reduce autoantibody production by specifically targeting autoreactive B cell populations (see Figure 4). B cell-depleting therapies, such as rituximab, have been investigated in a first clinical study in ME/CFS, although this first trial did not demonstrate clinical efficacy. More recent therapeutic concepts therefore focus on deeper B cell and plasma-cell depletion using monoclonal antibodies targeting CD19, CD20, or CD38 [125,126].

Another potential strategy is the modulation of regulatory T cells (Tregs) to influence autoantibody production. In a reduced uterine perfusion pressure (RUPP) rat model of preeclampsia, Ibrahim et al. induced endogenous Treg expansion using a CD28 superagonistic monoclonal antibody. The increase in Tregs was associated with reduced levels of AT1R autoantibodies, changes in inflammatory mediators, and a reduction in blood pressure [127]. These findings provide preclinical evidence that modulation of immune regulation may indirectly influence the production of functional GPCR autoantibodies.

## 7. Conclusions

Although the mechanisms underlying the development of GPCR-fAAbs are not yet fully understood, several processes, including infections, molecular mimicry, tissue damage, and dysregulation of immune tolerance, may contribute to their formation. By binding to extracellularly accessible regions of their target receptors, GPCR-fAAbs can modulate receptor function and intracellular signaling. The presence of GPCR-directed autoantibodies does not necessarily imply functional activity, and thus alone does not always lead to pathogenicity. However, for selected GPCR-fAAbs, increasing evidence supports a potential contribution to pathophysiological processes in certain diseases. The identification and functional characterization of GPCR-fAAbs in various disease contexts have also provided a basis for the development of targeted therapeutic strategies. Current and investigational approaches include the removal or neutralization of GPCR-fAAbs, modulation of autoantibody-producing immune cells, and pharmacological interference with their effects on target receptors. These strategies may complement existing symptomatic treatments and provide more targeted therapeutic options for selected patient populations. Further studies are needed to establish standardized and validated diagnostic approaches for GPCR-fAAbs suitable for routine clinical use, and to evaluate the efficacy and safety of targeted therapeutic approaches.

## Figures and Tables

**Figure 1:**
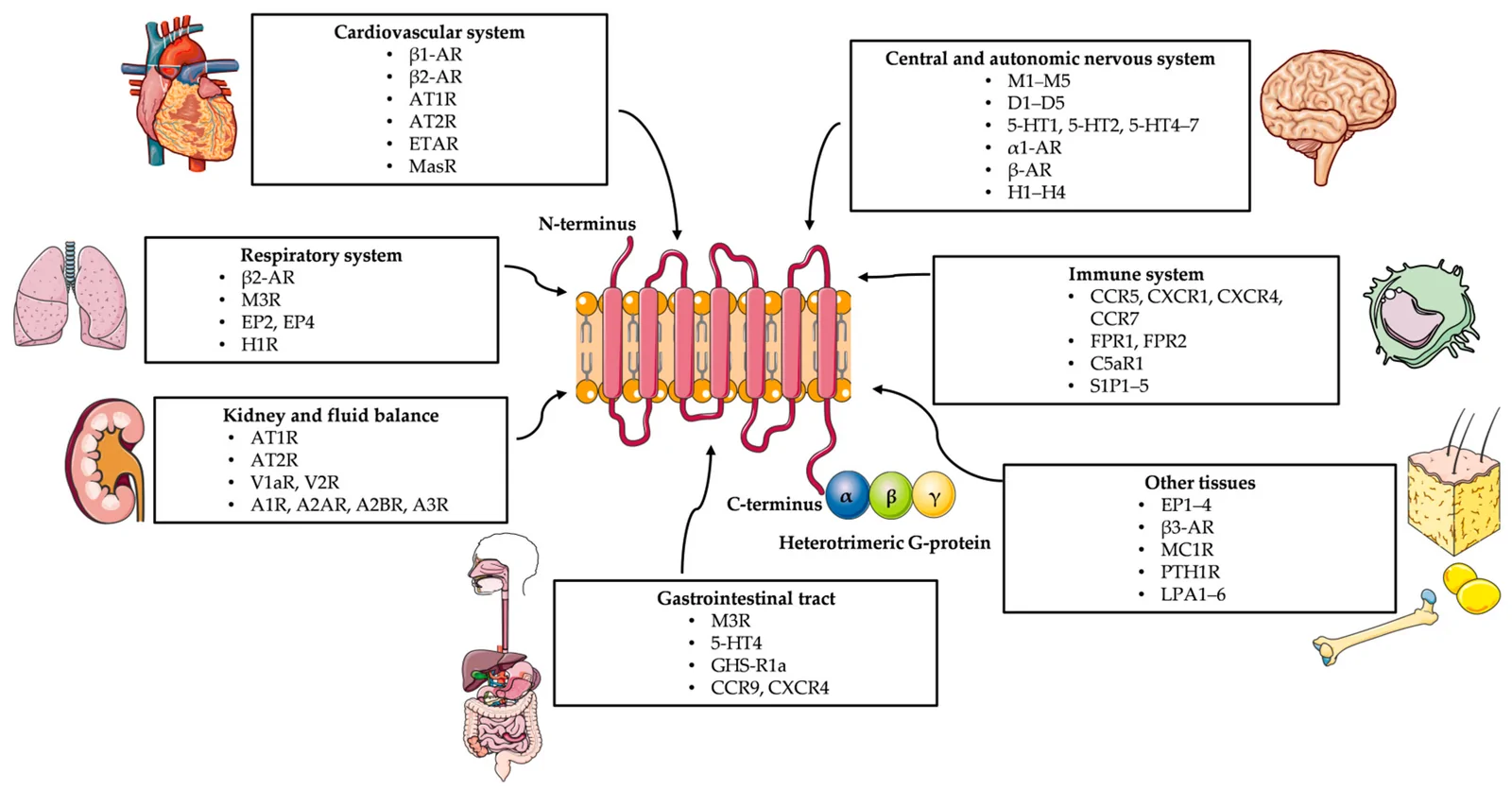
Distribution and physiological roles of major G-protein-coupled receptors (GPCRs) in human organs and tissues. Cardiovascular system: β1-AR (Beta 1-adrenergic receptor), β2-AR (Beta 2-adrenergic receptor), AT1R (Angiotensin II type 1 receptor), AT2R (Angiotensin II type 2 receptor), ETAR (Endothelin A receptor), MasR (Mas receptor) regulate cardiac function, vascular tone, and blood pressure. Central and autonomic nervous system: M1–M5 (Muscarinic acetylcholine receptor subtypes 1–5), D1–D5 (Dopamine receptor subtypes 1–5), 5-HT1, 5-HT2, 5-HT4–7 (Serotonin receptor subtypes 1, 2 and 4–7), α1-AR (Alpha 1-adrenergic receptor), β-AR (Beta-adrenergic receptor), and H1–H4 (Histamine receptor subtypes 1–4) contribute to cognition, mood, arousal, and autonomic control. Respiratory system: β2-AR, M3R (Muscarinic acetylcholine M3 receptor), EP2, EP4 (Prostaglandin E receptor subtypes 2 and 4), and H1R (Histamine receptor 1) regulate airway smooth muscle tone, secretion, and inflammatory responses. Immune system: CCR5 (C-C chemokine receptor 5), CCR7 (C-C chemokine receptor 7), CXCR1 (C-X-C chemokine receptor 1), CXCR4 (C-X-C chemokine receptor 4), FPR1, FPR2 (Formyl peptide receptor 1 and 2), C5aR1 (Complement component 5a receptor 1), S1P1–5 (Sphingosine-1-phosphate receptor subtypes 1–5) regulate leukocyte migration, activation, and inflammatory and immune responses. Kidney and fluid balance: AT1R and AT2R, V1aR (Vasopressin V1a receptor), V2R (Vasopressin V2 receptor), A1R (Adenosine A1 receptor), A2AR (Adenosine A2A receptor), A2BR (Adenosine A2B receptor), and A3R (Adenosine A3 receptor) contribute to renal function, fluid balance, and blood pressure regulation. Gastrointestinal tract: M3R, 5-HT4, CCR9 (C-C chemokine receptor 9), CXCR4, and GHS-R1a (Ghrelin receptor 1a) regulate intestinal motility, secretion, appetite, and mucosal immune surveillance. Other tissues: EP1–4 (Prostaglandin E receptor subtypes 1–4), β3-AR (Beta 3-adrenergic receptor), MC1R (Melanocortin 1 receptor), PTH1R (Parathyroid hormone 1 receptor), and LPA1–6 (Lysophosphatidic acid receptor subtypes 1–6) contribute to inflammation, metabolism, pigmentation, bone remodeling, and tissue homeostasis.

**Figure 2:**
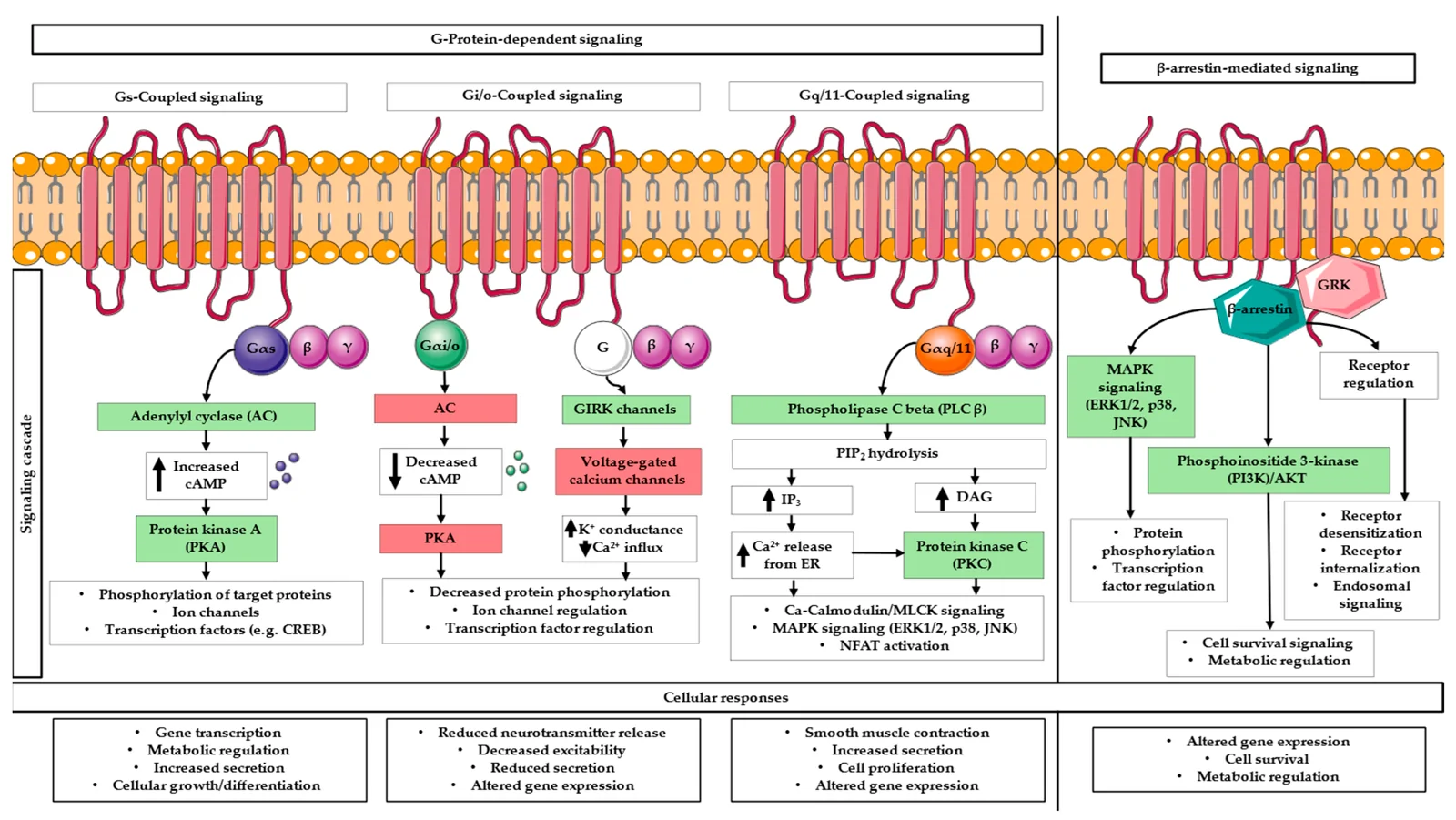
Overview of major GPCR-dependent signaling pathways. GPCR activation initiates G-protein-dependent signaling through Gs, Gi/o, or Gq/11 proteins and can additionally involve GRK-mediated receptor phosphorylation and β-arrestin recruitment. These pathways regulate intracellular second messengers, protein kinases, ion channels, receptor desensitization and internalization, and downstream cellular responses. Green boxes indicate activation and red boxes indicate inactivation. Abbreviations: AKT = Protein kinase B, cAMP = Cyclic adenosine monophosphate, CREB = cAMP response element-binding protein, DAG = Diacylglycerol, ER = Endoplasmic reticulum, ERK1/2 = Extracellular signal-regulated kinase 1/2, Gαi/o = G-protein alpha subunit i/o, Gαq/11 = G-protein alpha subunit q/11, Gαs = G-protein alpha subunit s, Gβγ = G-protein beta-gamma subunit complex, GIRK = G-protein-gated inwardly rectifying potassium channel, GRK = G-protein-coupled receptor kinase, IP_3_ = Inositol 1,4,5-trisphosphate, JNK = c-Jun N-terminal kinase, MAPK = Mitogen-activated protein kinase, MLCK = Myosin light-chain kinase, NFAT = Nuclear factor of activated T cells, PIP_2_ = Phosphatidylinositol 4,5-bisphosphate.

**Figure 3:**
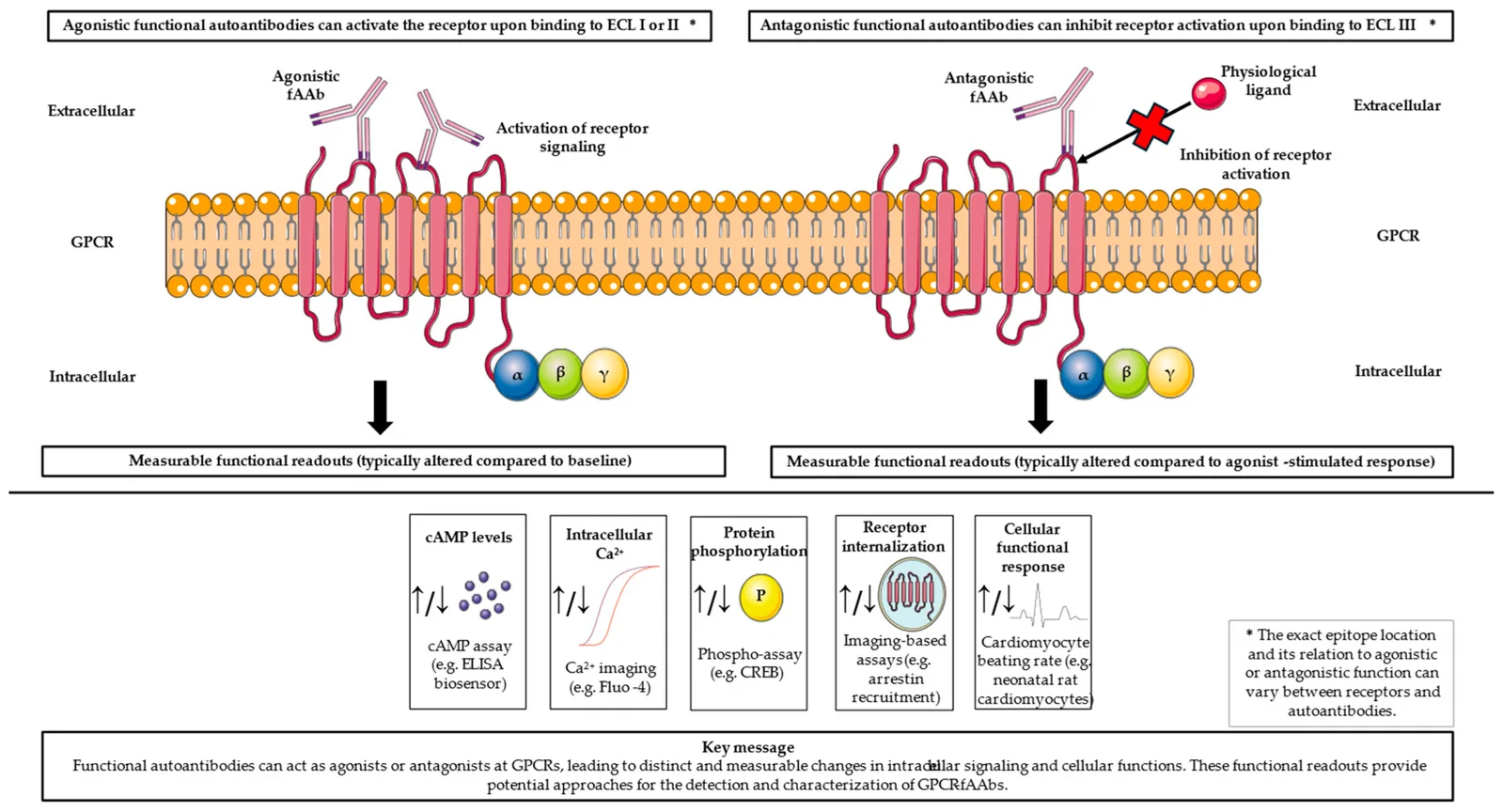
Summary of the functional effects of agonistic and antagonistic GPCR-targeting autoantibodies. Agonistic GPCR-fAAbs can activate GPCR signaling, whereas antagonistic GPCR-fAAbs can inhibit physiological ligand-induced receptor activation, resulting in measurable changes in intracellular signaling and cellular responses. Representative functional readouts include cAMP levels, intracellular Ca^2+^, protein phosphorylation, receptor internalization, and cellular functional responses. The direction of these responses may vary depending on the receptor, its G-protein coupling, and the measured readout. The arrows facing upwards or downwards indicate an increase or decrease in the values of the various functional parameters. The red cross indicates that the physiological ligand is unable to interact with the receptor. Abbreviations: cAMP = Cyclic adenosine monophosphate, CREB = cAMP response element-binding protein, ECL I-III = First, second, and third extracellular loop, ELISA = Enzyme-linked immunosorbent assay, fAAb = Functional autoantibody, GPCR = G-protein-coupled receptor.

**Figure 4:**
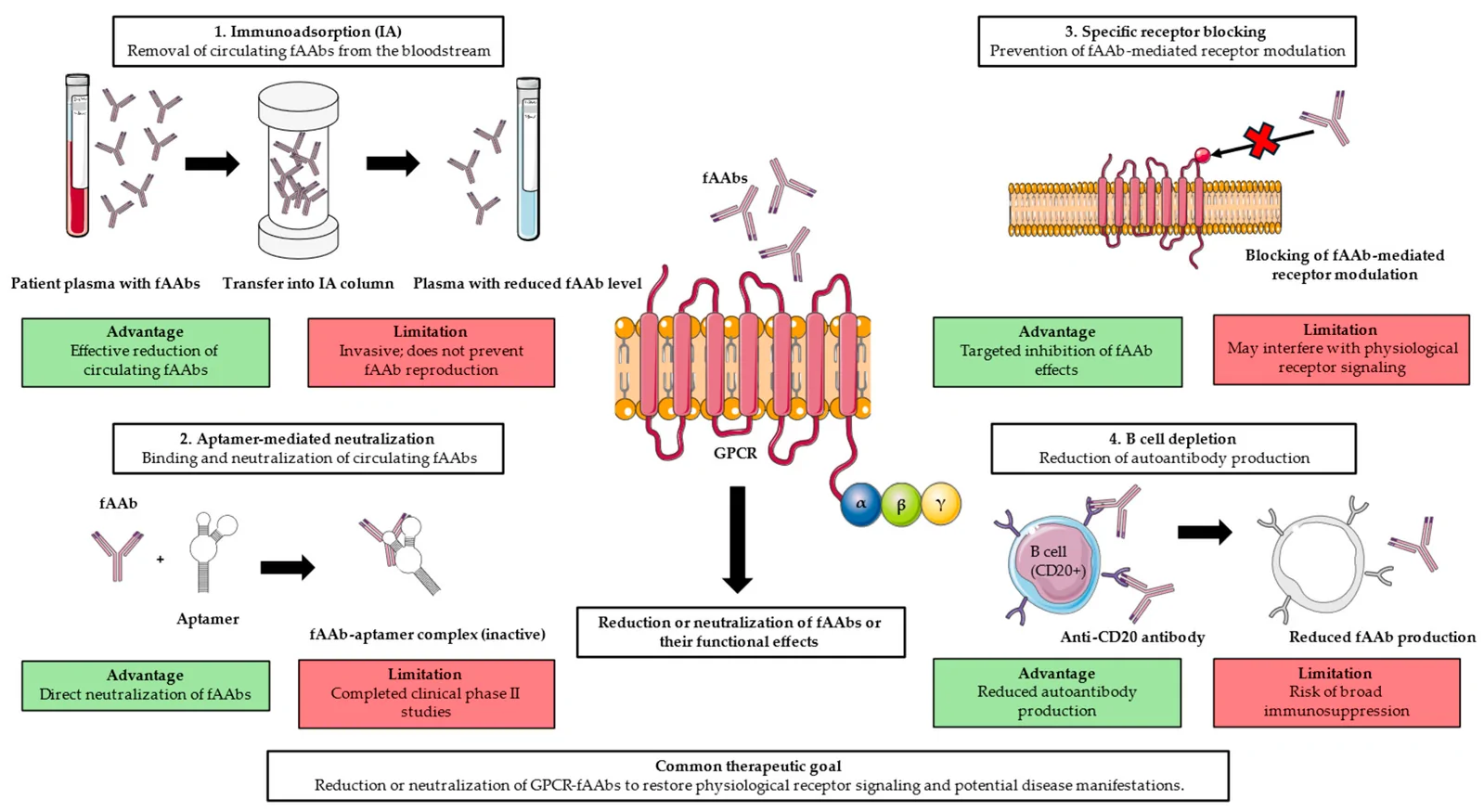
Potential therapeutic strategies targeting GPCR-fAAbs. Different therapeutic approaches may reduce or neutralize circulating GPCR-fAAbs or inhibit their functional effects. These strategies include IA for the removal of circulating autoantibodies, aptamer-mediated neutralization, specific receptor blocking to prevent GPCR-fAAb-mediated receptor modulation, and B cell depletion to reduce autoantibody production. Collectively, these approaches aim to reduce the pathogenic effects of GPCR-fAAbs and promote the restoration of physiological receptor signaling. The red cross indicates that the fAAb is unable to interact with the receptor. Abbreviations: fAAb = Functional autoantibody, GPCR = G-protein-coupled receptor.

**Table 1:** Functional GPCR-targeting autoantibodies associated with different diseases and clinical conditions. The table summarizes GPCR-fAAbs with reported functional effects on targeted receptor signaling, their proposed mechanism of action, the assay or detection method used for their characterization, and associated clinical manifestations. The detection methods were classified into three categories based on the type of experimental evidence they provide: B = Binding-based evidence: Detection of antibody binding or presence. F = in vitro functional evidence: Assessment of autoantibody-mediated effects on receptor signaling, cellular responses, or physiological functions. V = in vivo experimental evidence: Investigation of autoantibody-mediated effects in living organisms, including autoantibody-transfer models. Abbreviations: GABA(B)R = Gamma-aminobutyric acid type B receptor, M4R = Muscarinic acetylcholine M4 receptor, NOP = Nociceptin receptor, ME/CFS = Myalgic encephalomyelitis/chronic fatigue syndrome.

Category	Disease/Condition	Target Receptor(s)/G-Protein Coupling	Mechanism of Action	Detection Method	Associated Clinical Manifestation	Reference
Endocrinology	Graves’ disease	TSHR	Agonistic receptor activation	cAMP bioassay ^(F)^	Hyperthyroidism	Davies et al., 2020 [78]
Endocrinology	Autoimmune hypothyroidism	TSHR	Inhibition of TSH-induced receptor signaling	TSH-binding and cAMP inhibition assays ^(B,F)^	Hypothyroidism	Takasu & Matsushita, 2012 [79]
Infectious	Long-COVID	β2-AR, α1-AR, AT1R, M2-4R, MasR, ETAR and NOP	Functional receptor modulation	Cardiomyocyte bioassay/ELISA ^(B,F)^	Neurological and cardiovascular symptoms; impaired retinal microcirculation	Wallukat et al., 2021 [80], Seibert et al., 2023 [81], Szewczykowski et al., 2022 [49]
Cardiology	Idiopathic dilated cardiomyopathy	β1-AR and M2R	Chronic agonistic receptor stimulation	Cardiomyocyte bioassay and in vivo antibody-transfer model ^(F,V)^	Cardiac dysfunction and heart failure	Becker et al., 2017 [82], Wallukat et al., 2000 [83], Jahns et al., 2004 [38]
Cardiology	Postural tachycardia syndrome	α1-AR, β1-AR, β2-AR	Direct receptor activation and allosteric modulation	FRET-based reporter assay cAMP-dependent luciferase reporter assay ^(F)^	Tachycardia	Fedorowski et al., 2017 [34]
Cardiology	Orthostatic hypotension	β1-AR, β2-AR, M2R and M3R	Agonistic receptor activation	ELISA, cell-based functional assays, and vascular bioassay ^(B,F)^	Peripheral vasodilatation and orthostatic hypotension	Yu et al., 2012 [84], Li et al., 2012 [85]
Cardiology	Chagas cardiomyopathy	β1-AR and M2R	Chronic agonistic receptor stimulation	ELISA, immunofluorescence-based receptor-binding assay, and cardiac functional bioassays ^(B,F)^	Arrhythmias and cardiac dysfunction	Labovsky et al., 2007 [86], Hernández et al., 2003 [87]
Cardiology	Myocarditis	β1-AR	Agonistic receptor activation	Cardiomyocyte bioassay/ELISA ^(B,F)^	Cardiac dysfunction	Wallukat et al., 1991 [88], Seidel et al., 2022 [89]
Gynecology	Preeclampsia	AT1R and ETAR	Agonistic receptor stimulation	Cardiomyocyte bioassay with receptor-specific inhibition/neutralization ^(F)^	Hypertension and vascular dysfunction	Buttrup Larsen et al., 2018 [90], Velloso et al., 2016 [91]
Neurology	Autoimmune encephalitis	GABA(B)R	Functional disruption of receptor signaling	Cell-based immunofluorescence assay and whole-cell patch-clamp electrophysiology ^(B,F)^	Seizures and limbic encephalitis	Lancaster et al., 2010 [92], Nibber et al., 2017 [93]
Neurology	Alzheimer’s disease and vascular dementia	α1-AR, β2-AR, ETAR	Agonistic receptor activation	Cardiomyocyte bioassay/ELISA ^(B,F)^	Cognitive impairment and vascular dementia	Wallukat et al., 2018 [94], Karczewski et al., 2012 [95]
Ophthalmology	Open-angle glaucoma and ocular hypertension	β2-AR	Agonistic receptor activation	Cardiomyocyte bioassay ^(F)^	Elevated intraocular pressure	Hohberger et al., 2019 [96]
Respiratory disease	Bronchial asthma	β2-AR	Functional modulation of β2-adrenergic receptor activity	Cardiomyocyte bioassay; immunoprecipitation/radioligand-binding assay; cAMP assay in β2-AR-expressing cells ^(B,F)^	Bronchial asthma	Wallukat & Wollenberger, 1991 [97], Venter et al., 1980 [98], Turki & Liggett, 1995 [99]
Rheumatology	Sjögren’s syndrome	M3R	Inhibition of receptor signaling	Ca^2+^ imaging, patch-clamp electrophysiology, and peptide ELISA ^(B,F)^	Dryness of eye and mouth	Dawson et al., 2006 [41], Tsuboi et al., 2010 [100]
Rheumatology	Systemic sclerosis	AT1R, ETAR	Agonistic receptor activation	Solid-phase assay, immunoprecipitation, and endothelial cell-based functional assay ^(B,F)^	Vasculopathy, inflammation and fibrosis	Riemekasten et al., 2011 [101], Becker et al., 2014 [102]
Systemic disorders	ME/CFS	β2-AR	Agonistic receptor activation	β-arrestin recruitment and cAMP reporter assays ^(B,F)^	Fatigue and autonomic symptoms	Hartwig et al., 2020 [103]
Transplant-related	Renal allograft rejection	AT1R	Agonistic receptor activation	Cardiomyocyte bioassay ^(F)^	Vasculopathy, hypertension	Dragun et al., 2005 [25]

## Data Availability

No new data were created or analyzed in this study. Data sharing is not applicable to this article.

## Abbreviations

The following abbreviations are used in this manuscript:

A1R	Adenosine A1 receptor
A2AR	Adenosine A2A receptor
A2BR	Adenosine A2B receptor
A3R	Adenosine A3 receptor
AC	Adenylyl cyclase
AGPCR	Adhesion G-protein-coupled receptor
AKT	Protein kinase B
α1-AR	Alpha 1-adrenergic receptor
AR	Adrenergic receptor
AT1R	Angiotensin II type 1 receptor
AT2R	Angiotensin II type 2 receptor
β-AR	Beta-adrenergic receptor
β1-AR	Beta 1-adrenergic receptor
β1-fAAb	Functional autoantibodies against beta 1
β2-AR	Beta 2-adrenergic receptor
β2-fAAb	Functional autoantibodies against beta 2
β3-AR	Beta 3-adrenergic receptor
C5aR1	Complement component 5a receptor 1
cAMP	Cyclic adenosine monophosphate
CCR5	C-C chemokine receptor 5
CCR7	C-C chemokine receptor 7
CCR9	C-C chemokine receptor 9
CH2	Second constant domain of the heavy chain
CREB	cAMP response element-binding protein
CXCR1	C-X-C chemokine receptor 1
CXCR4	C-X-C chemokine receptor 4
D1–D5	Dopamine receptor subtypes 1–5
DAG	Diacylglycerol
DCM	Dilated cardiomyopathy
ECLs	Extracellular loops
ECL I-III	First, second, and third extracellular loop
ELISA	Enzyme-linked immunosorbent assay
EP1–4	Prostaglandin E receptor subtypes 1–4
ER	Endoplasmic reticulum
ERK1/2	Extracellular signal-regulated kinase 1/2
ETAR	Endothelin A receptor
fAAb	Functional autoantibody
Fab	Fragment antigen-binding
Fc	Fragment crystallizable
FPR1	Formyl peptide receptor 1
FPR2	Formyl peptide receptor 2
FRET	Fluorescence resonance energy transfer
GABA(B)R	Gamma-aminobutyric acid type B receptor
GAIN	GPCR autoproteolysis-inducing
Gαi/o	G-protein alpha subunit i/o
Gαq/11	G-protein alpha subunit q/11
Gαs	G-protein alpha subunit s
Gβγ	G-protein beta-gamma subunit complex
GDP	Guanosine diphosphate
GHS-R1a	Ghrelin receptor 1a
GIRK	G-protein-gated inwardly rectifying potassium channel
GPCR	G-protein-coupled receptor
GPCR-fAAbs	Functional autoantibodies directed against GPCRs
GRK	G-protein-coupled receptor kinase
GTP	Guanosine triphosphate
H1-H4	Histamine receptor subtypes 1–4
H1R	Histamine receptor 1
IA	Immunoadsorption
IgG	Immunoglobulin G
IP_3_	Inositol 1,4,5-trisphosphate
JNK	c-Jun N-terminal kinase
LPA1–6	Lysophosphatidic acid receptor subtypes 1–6
M1–M5	Muscarinic acetylcholine receptor subtypes 1–5
M2R	Muscarinic acetylcholine M2 receptor
M3R	Muscarinic acetylcholine M3 receptor
M4R	Muscarinic acetylcholine M4 receptor
MAPK	Mitogen-activated protein kinase
MasR	Mas receptor
MC1R	Melanocortin 1 receptor
ME/CFS	Myalgic encephalomyelitis/chronic fatigue syndrome
MLCK	Myosin light-chain kinase
NFAT	Nuclear factor of activated T cells
NOP	Nociceptin receptor
PI3K	Phosphoinositide 3-kinase
PIP_2_	Phosphatidylinositol 4,5-bisphosphate
PKA	Protein kinase A
PKC	Protein kinase C
PLC	Phospholipase C
PLCβ	Phospholipase C beta
POTS	Postural Orthostatic Tachycardia Syndrome
PTH1R	Parathyroid hormone 1 receptor
RET	Resonance energy transfer
RUPP	Reduced uterine perfusion pressure
S1P1–5	Sphingosine-1-phosphate receptor subtypes 1–5
Tregs	Regulatory T cells
TSHR	Thyroid-stimulating hormone receptor
V1aR	Vasopressin V1a receptor
V2R	Vasopressin V2 receptor
5-HT1–7	Serotonin receptor subtypes 1–7

## References

[1] Pierce K.L., Premont R.T., Lefkowitz R.J. (2002). Seven-transmembrane receptors. Nat. Rev. Mol. Cell Biol..

[2] Schulte G. (2024). International Union of Basic and Clinical Pharmacology CXV: The Class F of G Protein-Coupled Receptors. Pharmacol. Rev..

[3] Lagerström M.C., Schiöth H.B. (2008). Structural diversity of G protein-coupled receptors and significance for drug discovery. Nat. Rev. Drug Discov..

[4] Martinez-Marin D., Stroman G.C., Fulton C.J., Pruitt K. (2025). Frizzled receptors: Gatekeepers of Wnt signaling in development and disease. Front. Cell Dev. Biol..

[5] Lala T., Hall R.A. (2022). Adhesion G protein-coupled receptors: Structure, signaling, physiology, and pathophysiology. Physiol. Rev..

[6] Vizurraga A., Adhikari R., Yeung J., Yu M., Tall G.G. (2020). Mechanisms of adhesion G protein-coupled receptor activation. J. Biol. Chem..

[7] Rosenbaum D.M., Rasmussen S.G., Kobilka B.K. (2009). The structure and function of G-protein-coupled receptors. Nature.

[8] Insel P.A., Tang C.M., Hahntow I., Michel M.C. (2007). Impact of GPCRs in clinical medicine: Monogenic diseases, genetic variants and drug targets. Biochim. Biophys. Acta.

[9] Bornholz B., Wallukat G., Roggenbuck D., Schimke I. (2017). Autoantibodies Directed Against G-Protein-Coupled Receptors in Cardiovascular Diseases: Basics and Diagnostics. The Heart in Rheumatic, Autoimmune and Inflammatory Diseases.

[10] Becker N.P., Göttel P., Mueller J., Wallukat G., Schimke I. (2019). Functional autoantibody diseases: Basics and treatment related to cardiomyopathies. Front. Biosci. (Landmark Ed.).

[11] Fu M.L., Hoebeke J., Matsui S., Matoba M., Magnusson Y., Hedner T., Herlitz H., Hjalmarson A. (1994). Autoantibodies against cardiac G-protein-coupled receptors define different populations with cardiomyopathies but not with hypertension. Clin. Immunol. Immunopathol..

[12] Cabral-Marques O., Riemekasten G. (2017). Functional autoantibodies targeting G protein-coupled receptors in rheumatic diseases. Nat. Rev. Rheumatol..

[13] Insel P.A., Snead A., Murray F., Zhang L., Yokouchi H., Katakia T., Kwon O., Dimucci D., Wilderman A. (2012). GPCR expression in tissues and cells: Are the optimal receptors being used as drug targets?. Br. J. Pharmacol..

[14] Regard J.B., Sato I.T., Coughlin S.R. (2008). Anatomical profiling of G protein-coupled receptor expression. Cell.

[15] Chhatar S., Lal G. (2021). Role of adrenergic receptor signalling in neuroimmune communication. Curr. Res. Immunol..

[16] Sotzny F., Filgueiras I.S., Kedor C., Freitag H., Wittke K., Bauer S., Sepúlveda N., Mathias da Fonseca D.L., Baiocchi G.C., Marques A.H.C. (2022). Dysregulated autoantibodies targeting vaso- and immunoregulatory receptors in Post COVID Syndrome correlate with symptom severity. Front. Immunol..

[17] Sanders J., Chirgadze D.Y., Sanders P., Baker S., Sullivan A., Bhardwaja A., Bolton J., Reeve M., Nakatake N., Evans M. (2007). Crystal structure of the TSH receptor in complex with a thyroid-stimulating autoantibody. Thyroid.

[18] Lefkowitz R.J. (2013). A brief history of G-protein coupled receptors (Nobel Lecture). Angew. Chem. Int. Ed. Engl..

[19] Chiu M.L., Goulet D.R., Teplyakov A., Gilliland G.L. (2019). Antibody Structure and Function: The Basis for Engineering Therapeutics. Antibodies.

[20] Vidarsson G., Dekkers G., Rispens T. (2014). IgG Subclasses and Allotypes: From Structure to Effector Functions. Front. Immunol..

[21] Kawai A., Nagatomo Y., Yukino-Iwashita M., Nakazawa R., Taruoka A., Yumita Y., Takefuji A., Yasuda R., Toya T., Ikegami Y. (2023). β(1) Adrenergic Receptor Autoantibodies and IgG Subclasses: Current Status and Unsolved Issues. J. Cardiovasc. Dev. Dis..

[22] Nagatomo Y., Li D., Kirsop J., Borowski A., Thakur A., Tang W.H. (2016). Autoantibodies Specifically Against β1 Adrenergic Receptors and Adverse Clinical Outcome in Patients With Chronic Systolic Heart Failure in the β-Blocker Era: The Importance of Immunoglobulin G3 Subclass. J. Card. Fail..

[23] Jünemann A., Hohberger B., Rech J., Sheriff A., Fu Q., Schlötzer-Schrehardt U., Voll R.E., Bartel S., Kalbacher H., Hoebeke J. (2018). Agonistic Autoantibodies to the β2-Adrenergic Receptor Involved in the Pathogenesis of Open-Angle Glaucoma. Front. Immunol..

[24] Hohberger B., Schlötzer-Schrehardt U., Mardin C., Lämmer R., Munoz L., Kunze R., Herrmann M., Wallukat G. (2021). Inhibitory and Agonistic Autoantibodies Directed Against the β(2)-Adrenergic Receptor in Pseudoexfoliation Syndrome and Glaucoma. Front. Neurosci..

[25] Dragun D., Müller D.N., Bräsen J.H., Fritsche L., Nieminen-Kelhä M., Dechend R., Kintscher U., Rudolph B., Hoebeke J., Eckert D. (2005). Angiotensin II type 1-receptor activating antibodies in renal-allograft rejection. N. Engl. J. Med..

[26] Sterin-Borda L., Perez Leiros C., Wald M., Cremaschi G., Borda E. (1988). Antibodies to beta 1 and beta 2 adrenoreceptors in Chagas’ disease. Clin. Exp. Immunol..

[27] Staudt A., Eichler P., Trimpert C., Felix S.B., Greinacher A. (2007). Fc(gamma) receptors IIa on cardiomyocytes and their potential functional relevance in dilated cardiomyopathy. J. Am. Coll. Cardiol..

[28] Skiba M.A., Kruse A.C. (2021). Autoantibodies as Endogenous Modulators of GPCR Signaling. Trends Pharmacol. Sci..

[29] Hutchings C.J., Cseke G., Osborne G., Woolard J., Zhukov A., Koglin M., Jazayeri A., Pandya-Pathak J., Langmead C.J., Hill S.J. (2014). Monoclonal anti-β1-adrenergic receptor antibodies activate G protein signaling in the absence of β-arrestin recruitment. MAbs.

[30] Wölfel A., Sättele M., Zechmeister C., Nikolaev V.O., Lohse M.J., Boege F., Jahns R., Boivin-Jahns V. (2020). Unmasking features of the auto-epitope essential for β(1) -adrenoceptor activation by autoantibodies in chronic heart failure. ESC Heart Fail..

[31] Sanders J., Jeffreys J., Depraetere H., Evans M., Richards T., Kiddie A., Brereton K., Premawardhana L.D., Chirgadze D.Y., Núñez Miguel R. (2004). Characteristics of a human monoclonal autoantibody to the thyrotropin receptor: Sequence structure and function. Thyroid.

[32] Rees Smith B., Núñez Miguel R., Sanders J. (2025). The thyrotropin receptor structure and interactions with autoantibodies. Eur. Thyroid J..

[33] Jahns R., Boivin V., Krapf T., Wallukat G., Boege F., Lohse M.J. (2000). Modulation of beta1-adrenoceptor activity by domain-specific antibodies and heart failure-associated autoantibodies. J. Am. Coll. Cardiol..

[34] Fedorowski A., Li H., Yu X., Koelsch K.A., Harris V.M., Liles C., Murphy T.A., Quadri S.M.S., Scofield R.H., Sutton R. (2017). Antiadrenergic autoimmunity in postural tachycardia syndrome. Europace.

[35] Mohan M.L., Nagatomo Y., Saha P.P., Mukherjee S.D., Engelman T., Morales R., Hazen S.L., Tang W.H.W., Naga Prasad S.V. (2021). The IgG3 subclass of β1-adrenergic receptor autoantibodies is an endogenous biaser of β1AR signaling. Mol. Biol. Cell.

[36] Bornholz B., Weidtkamp-Peters S., Schmitmeier S., Seidel C.A., Herda L.R., Felix S.B., Lemoine H., Hescheler J., Nguemo F., Schäfer C. (2013). Impact of human autoantibodies on β1-adrenergic receptor conformation, activity, and internalization. Cardiovasc. Res..

[37] Cabral-Marques O., Schimke L.F., Moll G., Filgueiras I.S., Nóbile A.L., Adri A.S., do Vale F.Y.N., Usuda J.N., Corrêa Y.L.G., Albuquerque D. (2025). Advancing research on regulatory autoantibodies targeting GPCRs: Insights from the 5th international symposium. Autoimmun. Rev..

[38] Jahns R., Boivin V., Hein L., Triebel S., Angermann C.E., Ertl G., Lohse M.J. (2004). Direct evidence for a beta 1-adrenergic receptor-directed autoimmune attack as a cause of idiopathic dilated cardiomyopathy. J. Clin. Investig..

[39] Wheatley M., Wootten D., Conner M.T., Simms J., Kendrick R., Logan R.T., Poyner D.R., Barwell J. (2012). Lifting the lid on GPCRs: The role of extracellular loops. Br. J. Pharmacol..

[40] Steyaert J., Kobilka B.K. (2011). Nanobody stabilization of G protein-coupled receptor conformational states. Curr. Opin. Struct. Biol..

[41] Dawson L.J., Stanbury J., Venn N., Hasdimir B., Rogers S.N., Smith P.M. (2006). Antimuscarinic antibodies in primary Sjögren’s syndrome reversibly inhibit the mechanism of fluid secretion by human submandibular salivary acinar cells. Arthritis Rheum..

[42] Waterman S.A., Gordon T.P., Rischmueller M. (2000). Inhibitory effects of muscarinic receptor autoantibodies on parasympathetic neurotransmission in Sjögren’s syndrome. Arthritis Rheum..

[43] Lutz E.R., Wu A.A., Bigelow E., Sharma R., Mo G., Soares K., Solt S., Dorman A., Wamwea A., Yager A. (2014). Immunotherapy converts nonimmunogenic pancreatic tumors into immunogenic foci of immune regulation. Cancer Immunol. Res..

[44] Cabral-Marques O., Marques A., Giil L.M., De Vito R., Rademacher J., Günther J., Lange T., Humrich J.Y., Klapa S., Schinke S. (2018). GPCR-specific autoantibody signatures are associated with physiological and pathological immune homeostasis. Nat. Commun..

[45] Yu X., Wax J., Riemekasten G., Petersen F. (2023). Functional autoantibodies: Definition, mechanisms, origin and contributions to autoimmune and non-autoimmune disorders. Autoimmun. Rev..

[46] Johnson D., Jiang W. (2023). Infectious diseases, autoantibodies, and autoimmunity. J. Autoimmun..

[47] Fujinami R.S., von Herrath M.G., Christen U., Whitton J.L. (2006). Molecular mimicry, bystander activation, or viral persistence: Infections and autoimmune disease. Clin. Microbiol. Rev..

[48] Freitag H., Szklarski M., Lorenz S., Sotzny F., Bauer S., Philippe A., Kedor C., Grabowski P., Lange T., Riemekasten G. (2021). Autoantibodies to Vasoregulative G-Protein-Coupled Receptors Correlate with Symptom Severity, Autonomic Dysfunction and Disability in Myalgic Encephalomyelitis/Chronic Fatigue Syndrome. J. Clin. Med..

[49] Szewczykowski C., Mardin C., Lucio M., Wallukat G., Hoffmanns J., Schröder T., Raith F., Rogge L., Heltmann F., Moritz M. (2022). Long COVID: Association of Functional Autoantibodies against G-Protein-Coupled Receptors with an Impaired Retinal Microcirculation. Int. J. Mol. Sci..

[50] Ferrari I., Levin M.J., Wallukat G., Elies R., Lebesgue D., Chiale P., Elizari M., Rosenbaum M., Hoebeke J. (1995). Molecular mimicry between the immunodominant ribosomal protein P0 of Trypanosoma cruzi and a functional epitope on the human beta 1-adrenergic receptor. J. Exp. Med..

[51] Cabral-Marques O., Moll G., Catar R., Preuß B., Bankamp L., Pecher A.C., Henes J., Klein R., Kamalanathan A.S., Akbarzadeh R. (2023). Autoantibodies targeting G protein-coupled receptors: An evolving history in autoimmunity. Report of the 4th international symposium. Autoimmun. Rev..

[52] Gong X., Yang S.Y., Wang Z.Y., Tang M. (2024). The role of hypoxic microenvironment in autoimmune diseases. Front. Immunol..

[53] Hofmann S., Lucio M., Wallukat G., Hoffmanns J., Schröder T., Raith F., Szewczykowski C., Skornia A., Rech J., Schottenhamml J. (2025). Functional Autoantibodies Targeting G-Protein-Coupled Receptors and Their Clinical Phenotype in Patients with Long-COVID. Int. J. Mol. Sci..

[54] Wallukat G., Lakatos P., Steinhorst K., Flecks M., Hohberger B. (2026). An Explorative Approach to Examining the Role of Ischemia and Inflammation on the Function of Autoantibodies Against G Protein–Coupled Receptors and Their Corresponding Agonists. Int. J. Mol. Sci..

[55] Lukitsch I., Kehr J., Chaykovska L., Wallukat G., Nieminen-Kelhä M., Batuman V., Dragun D., Gollasch M. (2012). Renal ischemia and transplantation predispose to vascular constriction mediated by angiotensin II type 1 receptor-activating antibodies. Transplantation.

[56] Wallukat G., Homuth V., Fischer T., Lindschau C., Horstkamp B., Jüpner A., Baur E., Nissen E., Vetter K., Neichel D. (1999). Patients with preeclampsia develop agonistic autoantibodies against the angiotensin AT1 receptor. J. Clin. Investig..

[57] Thway T.M., Shlykov S.G., Day M.C., Sanborn B.M., Gilstrap L.C., Xia Y., Kellems R.E. (2004). Antibodies from preeclamptic patients stimulate increased intracellular Ca2+ mobilization through angiotensin receptor activation. Circulation.

[58] Zhu F., Sun Y.X., Liao Y.H., Wei Y.M., Chen M., Wang M., Zhou Z.H. (2008). Agonistic AT(1) receptor autoantibody increases in serum of patients with refractory hypertension and improves Ca(2+) mobilization in cultured rat vascular smooth muscle cells. Cell Mol. Immunol..

[59] Siddiqui A.H., Irani R.A., Blackwell S.C., Ramin S.M., Kellems R.E., Xia Y. (2010). Angiotensin receptor agonistic autoantibody is highly prevalent in preeclampsia: Correlation with disease severity. Hypertension.

[60] Wallukat G., Wollenberger A. (1987). Effects of the serum gamma globulin fraction of patients with allergic asthma and dilated cardiomyopathy on chronotropic beta adrenoceptor function in cultured neonatal rat heart myocytes. Biomed. Biochim. Acta.

[61] Wallukat G., Wollenberger A., Morwinski R., Pitschner H.F. (1995). Anti-beta 1-adrenoceptor autoantibodies with chronotropic activity from the serum of patients with dilated cardiomyopathy: Mapping of epitopes in the first and second extracellular loops. J. Mol. Cell. Cardiol..

[62] Wallukat G., Schimke I. (2014). Agonistic autoantibodies directed against G-protein-coupled receptors and their relationship to cardiovascular diseases. Semin. Immunopathol..

[63] Unal H., Jagannathan R., Karnik S.S. (2012). Mechanism of GPCR-directed autoantibodies in diseases. Adv. Exp. Med. Biol..

[64] Schmitz B., Garbsch R., Schäfer H., Bär C., Chatterjee S., Riemekasten G., Schulze-Forster K., Heidecke H., Schultheiß C., Binder M. (2026). Autonomic dysfunction and vasoregulation in long COVID-19 are linked to anti-GPCR autoantibodies. J. Allergy Clin. Immunol..

[65] Akbari A., Hadizadeh A., Islampanah M., Salavati Nik E., Atkin S.L., Sahebkar A. (2023). COVID-19, G protein-coupled receptor, and renin-angiotensin system autoantibodies: Systematic review and meta-analysis. Autoimmun. Rev..

[66] Jahns R., Boivin V., Siegmund C., Inselmann G., Lohse M.J., Boege F. (1999). Autoantibodies activating human beta1-adrenergic receptors are associated with reduced cardiac function in chronic heart failure. Circulation.

[67] Marmai A., Civieri G., Iop L., Martini M., Vadori M., Cozzi E., Tona F. (2026). Autoantibody Profiling in Cardiomyopathies: Toward Immune-Guided Risk Stratification and Therapy. J. Clin. Med..

[68] Sharma S., Checco J.W. (2021). Evaluating functional ligand-GPCR interactions in cell-based assays. Methods Cell Biol..

[69] Olivo P.D. (2023). Bioassays for thyrotropin receptor autoantibodies. Best Pract. Res. Clin. Endocrinol. Metab..

[70] Bankamp L., Preuß B., Pecher A.C., Beucke N., Henes J., Klein R. (2021). Functionally Active Antibodies to the Angiotensin II Type 1-Receptor Measured by a Luminometric Bioassay Do Not Correlate With Clinical Manifestations in Systemic Sclerosis: A Comparison With Antibodies to Vascular Receptors and Topoisomerase I Detected by ELISA. Front. Immunol..

[71] Bankamp L., Preuß B., Pecher A.-C., Vogel W., Henes J., Klein R. (2023). Functional autoantibodies in systemic sclerosis: Influence of autologous stem cell transplantation and correlation with clinical outcome. Rheumatology.

[72] Zhou Y., Meng J., Xu C., Liu J. (2021). Multiple GPCR Functional Assays Based on Resonance Energy Transfer Sensors. Front. Cell Dev. Biol..

[73] Li H., Zhang G., Forsythe E., Okamoto L.E., Yu X. (2022). Implications of Antimuscarinic Autoantibodies in Postural Tachycardia Syndrome. J. Cardiovasc. Transl. Res..

[74] Zheng C., Javitch J.A., Lambert N.A., Donthamsetti P., Gurevich V.V. (2023). In-Cell Arrestin-Receptor Interaction Assays. Curr. Protoc..

[75] Kharraziha I., Axelsson J., Ricci F., Di Martino G., Persson M., Sutton R., Fedorowski A., Hamrefors V. (2020). Serum Activity Against G Protein-Coupled Receptors and Severity of Orthostatic Symptoms in Postural Orthostatic Tachycardia Syndrome. J. Am. Heart Assoc..

[76] Riemekasten G., Petersen F., Heidecke H. (2020). What Makes Antibodies Against G Protein-Coupled Receptors so Special? A Novel Concept to Understand Chronic Diseases. Front. Immunol..

[77] Chen X., Kieselbach B., Krämer B.K., von Baehr V., Reichetzeder C., Hocher B. (2025). Associations between functional autoantibodies targeting GPCRs, antinuclear antibodies, and inflammatory cytokines TNF-α: A cross-sectional study of 19,810 individuals. Front. Immunol..

[78] Davies T.F., Andersen S., Latif R., Nagayama Y., Barbesino G., Brito M., Eckstein A.K., Stagnaro-Green A., Kahaly G.J. (2020). Graves’ disease. Nat. Rev. Dis. Primers.

[79] Takasu N., Matsushita M. (2012). Changes of TSH-Stimulation Blocking Antibody (TSBAb) and Thyroid Stimulating Antibody (TSAb) Over 10 Years in 34 TSBAb-Positive Patients with Hypothyroidism and in 98 TSAb-Positive Graves’ Patients with Hyperthyroidism: Reevaluation of TSBAb and TSAb in TSH-Receptor-Antibody (TRAb)-Positive Patients. J. Thyroid Res..

[80] Wallukat G., Hohberger B., Wenzel K., Fürst J., Schulze-Rothe S., Wallukat A., Hönicke A.S., Müller J. (2021). Functional autoantibodies against G-protein coupled receptors in patients with persistent Long-COVID-19 symptoms. J. Transl. Autoimmun..

[81] Seibert F.S., Stervbo U., Wiemers L., Skrzypczyk S., Hogeweg M., Bertram S., Kurek J., Anft M., Westhoff T.H., Babel N. (2023). Severity of neurological Long-COVID symptoms correlates with increased level of autoantibodies targeting vasoregulatory and autonomic nervous system receptors. Autoimmun. Rev..

[82] Becker N.P., Müller J., Göttel P., Wallukat G., Schimke I. (2017). Cardiomyopathy—An approach to the autoimmune background. Autoimmun. Rev..

[83] Wallukat G., Nissen E., Morwinski R., Müller J. (2000). Autoantibodies against the beta- and muscarinic receptors in cardiomyopathy. Herz.

[84] Yu X., Stavrakis S., Hill M.A., Huang S., Reim S., Li H., Khan M., Hamlett S., Cunningham M.W., Kem D.C. (2012). Autoantibody activation of beta-adrenergic and muscarinic receptors contributes to an “autoimmune” orthostatic hypotension. J. Am. Soc. Hypertens..

[85] Li H., Kem D.C., Reim S., Khan M., Vanderlinde-Wood M., Zillner C., Collier D., Liles C., Hill M.A., Cunningham M.W. (2012). Agonistic autoantibodies as vasodilators in orthostatic hypotension: A new mechanism. Hypertension.

[86] Labovsky V., Smulski C.R., Gómez K., Levy G., Levin M.J. (2007). Anti-beta1-adrenergic receptor autoantibodies in patients with chronic Chagas heart disease. Clin. Exp. Immunol..

[87] Hernández C.C., Barcellos L.C., Giménez L.E., Cabarcas R.A., Garcia S., Pedrosa R.C., Nascimento J.H., Kurtenbach E., Masuda M.O., Campos de Carvalho A.C. (2003). Human chagasic IgGs bind to cardiac muscarinic receptors and impair L-type Ca2+ currents. Cardiovasc. Res..

[88] Wallukat G., Morwinski M., Kowal K., Förster A., Boewer V., Wollenberger A. (1991). Autoantibodies against the beta-adrenergic receptor in human myocarditis and dilated cardiomyopathy: Beta-adrenergic agonism without desensitization. Eur. Heart J..

[89] Seidel F., Scheibenbogen C., Heidecke H., Opgen-Rhein B., Pickardt T., Klingel K., Berger F., Messroghli D., Schubert S. (2022). Compensatory Upregulation of Anti-Beta-Adrenergic Receptor Antibody Levels Might Prevent Heart Failure Presentation in Pediatric Myocarditis. Front. Pediatr..

[90] Buttrup Larsen S., Wallukat G., Schimke I., Sandager A., Tvilum Christensen T., Uldbjerg N., Tørring N. (2018). Functional autoantibodies against Endothelin-1 receptor type A and Angiotensin II receptor type 1 in patients with preeclampsia. Pregnancy Hypertens..

[91] Velloso E.P., Pimentel R.L., Braga J.F., Cabral A.C., Reis Z.S., Bader M., Santos R.A., Wallukat G. (2016). Identification of a Novel Agonist-Like Autoantibody in Preeclamptic Patients. Am. J. Hypertens..

[92] Lancaster E., Lai M., Peng X., Hughes E., Constantinescu R., Raizer J., Friedman D., Skeen M.B., Grisold W., Kimura A. (2010). Antibodies to the GABA(B) receptor in limbic encephalitis with seizures: Case series and characterisation of the antigen. Lancet Neurol..

[93] Nibber A., Mann E.O., Pettingill P., Waters P., Irani S.R., Kullmann D.M., Vincent A., Lang B. (2017). Pathogenic potential of antibodies to the GABA(B) receptor. Epilepsia Open.

[94] Wallukat G., Prüss H., Müller J., Schimke I. (2018). Functional autoantibodies in patients with different forms of dementia. PLoS ONE.

[95] Karczewski P., Hempel P., Kunze R., Bimmler M. (2012). Agonistic autoantibodies to the α(1) -adrenergic receptor and the β(2) -adrenergic receptor in Alzheimer’s and vascular dementia. Scand. J. Immunol..

[96] Hohberger B., Kunze R., Wallukat G., Kara K., Mardin C.Y., Lämmer R., Schlötzer-Schrehardt U., Hosari S., Horn F., Munoz L. (2019). Autoantibodies Activating the β2-Adrenergic Receptor Characterize Patients With Primary and Secondary Glaucoma. Front. Immunol..

[97] Wallukat G., Wollenberger A. (1991). Autoantibodies to beta 2-adrenergic receptors with antiadrenergic activity from patients with allergic asthma. J. Allergy Clin. Immunol..

[98] Venter J.C., Fraser C.M., Harrison L.C. (1980). Autoantibodies to beta 2-adrenergic receptors: A possible cause of adrenergic hyporesponsiveness in allergic rhinitis and asthma. Science.

[99] Turki J., Liggett S.B. (1995). Receptor-specific functional properties of beta 2-adrenergic receptor autoantibodies in asthma. Am. J. Respir. Cell Mol. Biol..

[100] Tsuboi H., Matsumoto I., Wakamatsu E., Nakamura Y., Iizuka M., Hayashi T., Goto D., Ito S., Sumida T. (2010). New epitopes and function of anti-M3 muscarinic acetylcholine receptor antibodies in patients with Sjögren’s syndrome. Clin. Exp. Immunol..

[101] Riemekasten G., Philippe A., Näther M., Slowinski T., Müller D.N., Heidecke H., Matucci-Cerinic M., Czirják L., Lukitsch I., Becker M. (2011). Involvement of functional autoantibodies against vascular receptors in systemic sclerosis. Ann. Rheum. Dis..

[102] Becker M.O., Kill A., Kutsche M., Guenther J., Rose A., Tabeling C., Witzenrath M., Kühl A.A., Heidecke H., Ghofrani H.A. (2014). Vascular receptor autoantibodies in pulmonary arterial hypertension associated with systemic sclerosis. Am. J. Respir. Crit. Care Med..

[103] Hartwig J., Sotzny F., Bauer S., Heidecke H., Riemekasten G., Dragun D., Meisel C., Dames C., Grabowski P., Scheibenbogen C. (2020). IgG stimulated β2 adrenergic receptor activation is attenuated in patients with ME/CFS. Brain Behav. Immun. Health.

[104] Binda M., Moccaldi B., Civieri G., Cuberli A., Doria A., Tona F., Zanatta E. (2024). Autoantibodies Targeting G-Protein-Coupled Receptors: Pathogenetic, Clinical and Therapeutic Implications in Systemic Sclerosis. Int. J. Mol. Sci..

[105] Zhang J., Xu H., Li Z., Feng F., Wang S., Li Y. (2025). Frequency of autoantibodies and their associated clinical characteristics and outcomes in patients with dilated cardiomyopathy: A systematic review and meta-analysis. Autoimmun. Rev..

[106] Felix S.B., Staudt A., Landsberger M., Grosse Y., Stangl V., Spielhagen T., Wallukat G., Wernecke K.D., Baumann G., Stangl K. (2002). Removal of cardiodepressant antibodies in dilated cardiomyopathy by immunoadsorption. J. Am. Coll. Cardiol..

[107] Müller J., Wallukat G., Dandel M., Bieda H., Brandes K., Spiegelsberger S., Nissen E., Kunze R., Hetzer R. (2000). Immunoglobulin Adsorption in Patients With Idiopathic Dilated Cardiomyopathy. Circulation.

[108] Mobini R., Staudt A., Felix S.B., Baumann G., Wallukat G., Deinum J., Svensson H., Hjalmarson A., Fu M. (2003). Hemodynamic improvement and removal of autoantibodies against beta1-adrenergic receptor by immunoadsorption therapy in dilated cardiomyopathy. J. Autoimmun..

[109] Dandel M., Wallukat G., Englert A., Lehmkuhl H.B., Knosalla C., Hetzer R. (2012). Long-term benefits of immunoadsorption in β(1)-adrenoceptor autoantibody-positive transplant candidates with dilated cardiomyopathy. Eur. J. Heart Fail..

[110] Felix S.B., Böhm M., Braun-Dullaeus R.C., Caforio A.L.P., Erdmann E., Grabmaier U., Groß S., Klingel K., Knebel F., Lehnert K. (2026). Immunoadsorption in dilated cardiomyopathy: The IASO-DCM trial. Eur. Heart J..

[111] Dörr M., Böhm M., Erdmann E., Groß S., Mahabadi A.A., Nauck M., Nickening G., Schultheiss H.P., Staudt A., Werdan K. (2024). Multicentre, randomized, double-blind, prospective study on the effects of ImmunoAdSorptiOn on cardiac function in patients with Dilated CardioMyopathy (IASO-DCM): Rationale and design. Eur. J. Heart Fail..

[112] Tölle M., Freitag H., Antelmann M., Hartwig J., Schuchardt M., van der Giet M., Eckardt K.-U., Grabowski P., Scheibenbogen C. (2020). Myalgic Encephalomyelitis/Chronic Fatigue Syndrome: Efficacy of Repeat Immunoadsorption. J. Clin. Med..

[113] Haberland A., Holtzhauer M., Schlichtiger A., Bartel S., Schimke I., Müller J., Dandel M., Luppa P.B., Wallukat G. (2016). Aptamer BC 007—A broad spectrum neutralizer of pathogenic autoantibodies against G-protein-coupled receptors. Eur. J. Pharmacol..

[114] Weisshoff H., Krylova O., Nikolenko H., Düngen H.D., Dallmann A., Becker S., Göttel P., Müller J., Haberland A. (2020). Aptamer BC 007—Efficient binder of spreading-crucial SARS-CoV-2 proteins. Heliyon.

[115] Wallukat G., Müller J., Haberland A., Berg S., Schulz A., Freyse E.J., Vetter R., Salzsieder E., Kreutz R., Schimke I. (2016). Aptamer BC007 for neutralization of pathogenic autoantibodies directed against G-protein coupled receptors: A vision of future treatment of patients with cardiomyopathies and positivity for those autoantibodies. Atherosclerosis.

[116] Haberland A., Wallukat G., Dahmen C., Kage A., Schimke I. (2011). Aptamer neutralization of beta1-adrenoceptor autoantibodies isolated from patients with cardiomyopathies. Circ. Res..

[117] Becker N.P., Haberland A., Wenzel K., Göttel P., Wallukat G., Davideit H., Schulze-Rothe S., Hönicke A.S., Schimke I., Bartel S. (2020). A Three-Part, Randomised Study to Investigate the Safety, Tolerability, Pharmacokinetics and Mode of Action of BC 007, Neutraliser of Pathogenic Autoantibodies Against G-Protein Coupled Receptors in Healthy, Young and Elderly Subjects. Clin. Drug Investig..

[118] Hohberger B., Ganslmayer M., Harrer T., Kruse F., Maas S., Borst T., Heimke-Brinck R., Stog A., Knauer T., Rühl E. (2025). Safety, tolerability and clinical effects of rovunaptabin, also known as BC007 on fatigue and quality of life in patients with Post-COVID syndrome (reCOVer): A prospective, exploratory, placebo-controlled, double-blind, randomised phase IIa clinical trial (RCT). EClinicalMedicine.

[119] Wohlrab F., Eltity M., Ufer F., Paul F., Scheibenbogen C., Bellmann-Strobl J. (2025). Autoantibody targeting therapies in post COVID syndrome and myalgic encephalomyelitis/chronic fatigue syndrome. Expert. Opin. Biol. Ther..

[120] Magnusson Y., Wallukat G., Waagstein F., Hjalmarson A., Hoebeke J. (1994). Autoimmunity in idiopathic dilated cardiomyopathy. Characterization of antibodies against the beta 1-adrenoceptor with positive chronotropic effect. Circulation.

[121] Müller J., Wallukat G., Schimke I. (2017). Autoantibodies Directed Against the β(1)-Adrenergic Receptor in Patients With Dilated Cardiomyopathy. J. Am. Coll. Cardiol..

[122] Zhu G., Niu G., Chen X. (2015). Aptamer-Drug Conjugates. Bioconjug. Chem..

[123] Andrieu J., Re F., Russo L., Nicotra F. (2019). Phage-displayed peptides targeting specific tissues and organs. J. Drug Target..

[124] Scheibenbogen C., Bellmann-Strobl J.T., Heindrich C., Wittke K., Stein E., Franke C., Prüss H., Preßler H., Machule M.-L., Audebert H. (2023). Fighting Post-COVID and ME/CFS—Development of curative therapies. Front. Med..

[125] Fluge Ø., Tronstad K.J., Mella O. (2021). Pathomechanisms and possible interventions in myalgic encephalomyelitis/chronic fatigue syndrome (ME/CFS). J. Clin. Investig..

[126] Fluge Ø., Rekeland I.G., Sørland K., Alme K., Risa K., Bruland O., Tronstad K.J., Mella O. (2025). Plasma cell targeting with the anti-CD38 antibody daratumumab in myalgic encephalomyelitis/chronic fatigue syndrome-a clinical pilot study. Front. Med..

[127] Ibrahim T., Przybyl L., Harmon A.C., Amaral L.M., Faulkner J.L., Cornelius D.C., Cunningham M.W., Hünig T., Herse F., Wallukat G. (2017). Proliferation of endogenous regulatory T cells improve the pathophysiology associated with placental ischaemia of pregnancy. Am. J. Reprod. Immunol..

